# Image dataset of common benthic foraminiferal taxa in the North Atlantic seafloor surface sediments (59.5°N transect) between the Labrador sea and Faeroe-Shetland sill

**DOI:** 10.1016/j.dib.2019.104554

**Published:** 2019-09-26

**Authors:** Anna Tikhonova, Sofia Merenkova, Sergei Korsun, Alexander Matul

**Affiliations:** a Shirshov Institute of Oceanology, Russian Academy of Sciences, Russia; b Lomonosov Moscow State University, Russia

**Keywords:** Benthic foraminifera, Biodiversity, North Atlantic, Micropaleontology, Microphotograph

## Abstract

The article presents a microphotograph dataset of 106 taxa of benthic foraminifera (BF) from surface sediments collected at 26 grab station during RV *Akademik Ioffe* 51st cruise (summer 2016). The selected stations are located along a 59.5°N transect in the North Atlantic. This is the key area of climate control, where the interaction of warm surface and cold deep water masses takes place, which affects the operation of the global oceanic thermohaline circulation. The analysis of the benthic foraminiferal assemblages allows us to describe the properties of bottom water mass, to draw conclusions about the environmental parameters at the bottom. This photo dataset will facilitate the identification of species of BF, and thus will accelerate the routine process of micropaleontological analysis and the subsequent reconstruction of environmental conditions.

**Table:** Specifications Table

Subject	Earth and Planetary Sciences Paleontology
Specific subject area	Benthic Foraminifera Marine bottom sediments
Type of data	Tables Figure Phototables
How data were acquired	Samples were collected during the expedition to the North Atlantic in the summer 2016, using grab sampler; benthic foraminifera were identified to the species level and counted under the microscope ZEISS Stemi 508; photos were made using a Nikon microscope SMZ25 equipped with a Nikon camera DS-Fi3 and NIS-Elements D software. Microphotograph tables were made in the computer program CorelDRAW12.
Data format	Microphotograph tables of benthic foraminifera– raw data; figure with map of station– raw data; table with station list and their coordinates, and total counts of individual species– raw data.
Parameters for data collection	Standard laboratory processing of samples for micro-palaeontological analysis of bottom sediments using sieves of mesh size 63 μm.
Description of data collection	The samples were identified under the stereomicroscope, BF tests were counted and selected for the microphotography according the typical description of species.
Data source location	The North Atlantic at 59.5° N transect, the Labrador Sea and the Faeroe-Shetland sill (GPS coordinates are provided in the table 1).
Data accessibility	The data are available with this article

**Table:** 

**Value of the Data** • This data presents reflects changes in the BF assemblages in the key area of the global ocean termohaline circulation • The data are a comprehensive image collection representing common species of BF in the North Atlantic; the gallery can be used as a micropaleontological atlas, which helps to identify BF. Data may be useful in micropaleontology, taxonomy, paleoceanography. • The data are valuable for understanding the diversity of BF in the high northern latitudes of the Atlantic ocean.

## 1 Data

**Fig. 1:**
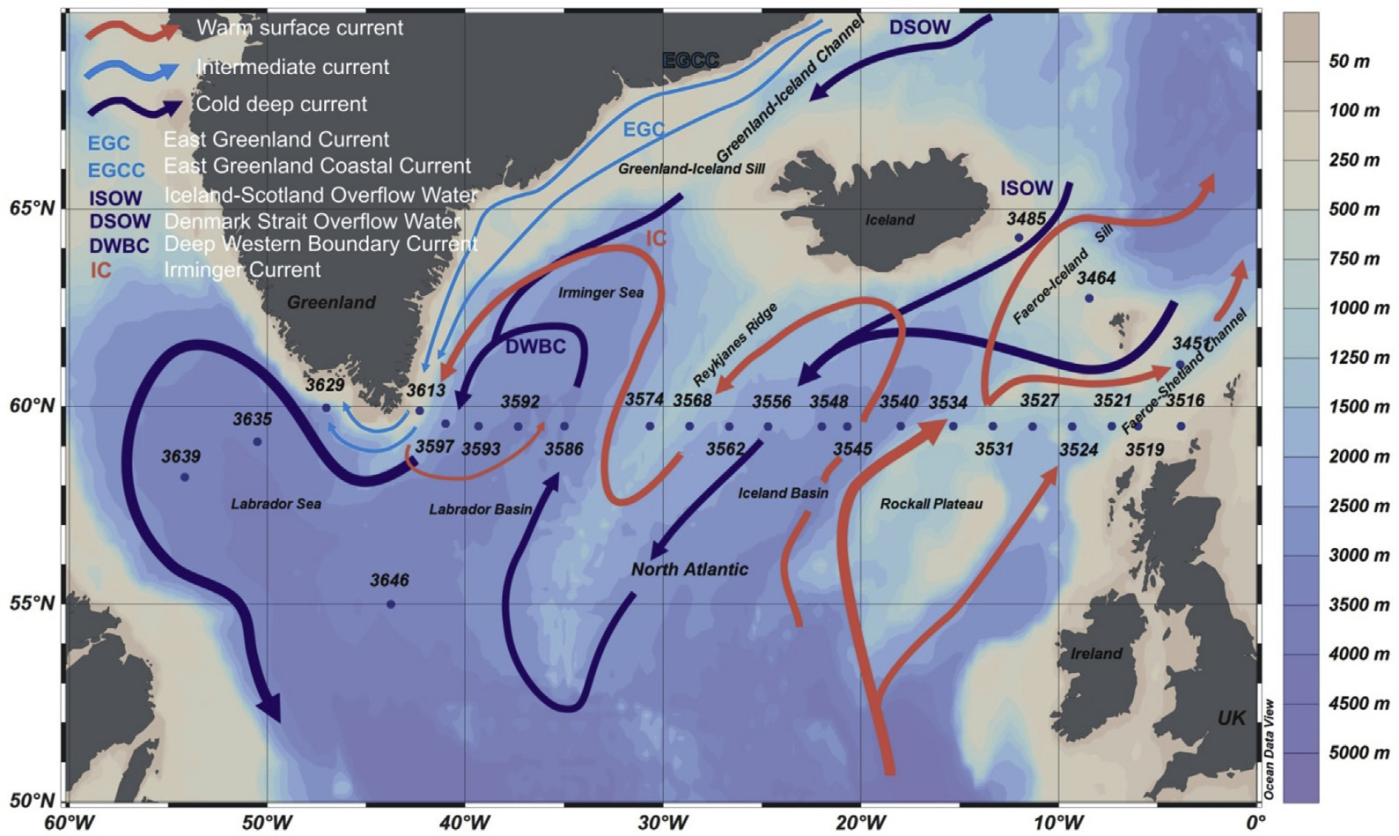
Map of the North Atlantic showing the location of stations. Red arrows denote warm surface currents, and blue arrows denote cold deep currents [10,11].
- station list with geographic coordinates, sampling depth, and number of tests counted in the sample (Table 1);

In this article, we present:

- map of the North Atlantic showing location of the research stations as well as warm surface currents and cold deep currents (Fig. 1);

**Table 1:** Station list with geographic coordinates, water depths, and the number of foraminiferal specimens counted in the sample.

Station No.	Depth (m)	Lat. (N)	Long. (W)	Total BF tests counted per 1 sample
AI3646	3346	55.0021°	−43.7569°	158
AI3639	3393	58.2180°	−54.1686°	368
AI3635	3477	59.1116°	−50.5108°	151
AI3629	2389	59.9664°	−47.0353°	152
AI3613	328	59.8962°	−42.3150°	399
AI3597	2399	59.5627°	−41.0077°	184
AI3593	2922	59.5028°	−39.3311°	193
AI3592	3156	59.4926°	−37.3242°	208
AI3586	3064	59.5039°	−34.9820°	159
AI3574	1531	59.5062°	−30.6606°	221
AI3568	1694	59.5009°	−28.6663°	199
AI3562	2237	59.4988°	−26.6601°	185
AI3556	2512	59.4977°	−24.7053°	157
AI3548	2740	59.4931°	−21.9865°	203
AI3545	2825	59.4984°	−20.6946°	160
AI3540	2182	59.5004°	−18.0003°	224
AI3534	1519	59.5028°	−15.3330°	294
AI3531	1291	59.5009°	−13.3339°	168
AI3527	1611	59.4991°	−11.3338°	209
AI3524	1468	59.4988°	−9.3336°	237
AI3521	1051	59.5001°	−7.3344°	323
AI3519	137	59.4996°	−6.0041°	295
AI3516	158	59.5001°	−3.8321°	422
AI3451	1150	61.0679°	−3.8666°	186
AI3464	484	62.7388°	−8.4601°	321
AI3485	567	64.0252°	−12.6182°	204
- list of BF taxa (Table 2);

**Table 2:** List of benthic foraminiferal taxa.

Benthic foraminiferal genera and species
*Adercotryma glomeratum* (Brady, 1878)
*Alabaminella weddellensis* (Earland, 1936)
*Amphicoryna scalaris* (Batsch, 1791)
*Astrononion gallowayi* Loeblich & Tappan, 1953 = *Astrononion hamadaense* Asano, 1950
*Astrononion stelligerum* (d'Orbigny, 1839)
*Bolivina earlandi* Parr, 1950
*Bolivina pseudoplicata* Heron-Allen & Earland, 1930
*Bolivina pygmaea* (Brady, 1881)
*Bolivina* sp. d'Orbigny, 1839
*Brizalina subspinescens* (Cushman, 1922)
*Bulimina marginata* d'Orbigny, 1826
*Bulimina mexicana* Cushman, 1922 = *Bulimina striata* d'Orbigny in Guérin-Méneville, 1832
*Carpenteria balaniformis* Gray, 1858
*Cassidulina carinata* Silvestri, 1896
*Cassidulina laevigata* d'Orbigny, 1826
*Cassidulina reniforme* Nørvang, 1945
*Cassidulina teretis* Tappan, 1951
*Cassidulinoides bradyi* (Norman, 1881) = *Evolvocassidulina bradyi* (Norman, 1881)
*Cibicides refulgens* Montfort, 1808
*Cibicidoides bradyi* (Trauth, 1918)
*Cibicidoides lobatulus* (Walker & Jacob, 1798)
*Cibicidoides pachyderma* (Rzehak, 1886)
*Cibicidoides pseudoungeriana* (Cushman, 1922)
*Cibicidoides* sp. Thalmann, 1939
*Cibicidoides wuellerstorfi* (Schwager, 1866)
*Cribrostomoides subglobosus* (Cushman, 1910)
*Cyclammina pusilla* Brady, 1881
*Cystammina pauciloculata* (Brady, 1879)
*Eggerella bradyi* (Cushman, 1911)
*Elphidium excavatum* subsp. *clavatum* Cushman, 1930
*Elphidium incertum* (Williamson, 1858) = *Cribroelphidium incertum* Williamson, 1858
*Elphidium* sp. Montfort, 1808
*Elphidium subarcticum* Cushman, 1944
*Epistominella exigua* (Brady, 1884)
*Fissurina lacunata* (Burrows & Holland, 1895) = *Seguenzaella lacunata* (Burrows & Holland, 1895)
*Fissurina* sp. Reuss, 1850
*Fissurina squamosoalata* (Brady, 1881) = *Vasicostella squamosoalata* (Brady, 1881)
*Fissurina staphyllearia* Schwager, 1866
*Fursenkoina complanata* (Egger, 1893)
*Fursenkoina fusiformis* (Williamson, 1858) = *Stainforthia fusiformis* (Williamson, 1858)
*Fursenkoina pauciloculata* (Brady, 1884)
*Gavelinopsis praegeri* (Heron-Allen & Earland, 1913)
*Globocassidulina subglobosa* (Brady, 1881)
*Gyroidina orbicularis* d'Orbigny in Parker, Jones & Brady, 1865
*Gyroidina* sp. d'Orbigny, 1826
*Hansenisca soldanii* (d'Orbigny, 1826)
*Hansenisca* sp. Loeblich & Tappan, 1987
*Haynesina orbicularis* (Brady, 1881)
*Hoeglundina elegans* (d'Orbigny, 1826)
*Hyalinea balthica* (Schröter in Gmelin, 1791)
*Ioanella tumidula* (Brady, 1884)
*Islandiella helenae* Feyling-Hanssen & Buzas, 1976
*Karreriella bradyi* (Cushman, 1911)
*Karrerulina conversa* (Grzybowski, 1901)
*Karrerulina* sp. Finlay, 1940
*Lagenammina difflugiformis* (Brady, 1879)
*Lagenosolenia incomposita* Patterson & Pettis, 1986 = *Fissurina incomposita* (Patterson & Pettis, 1986)
*Laticarinina pauperata* (Parker & Jones, 1865)
*Lenticulina gibba* (d'Orbigny, 1839)
*Melonis barleeanus* (Williamson, 1858)
*Melonis pompilioides* (Fichtel & Moll, 1798)
*Melonis* sp. Montfort, 1808
*Miliolinella subrotunda* (Montagu, 1803)
*Nonionella auricula* Heron-Allen & Earland, 1930
*Nonionella* sp. Rhumbler, 1949
*Nonionella turgida* (Williamson, 1858) = *Nonionoides turgidus* (Williamson, 1858)
*Nonionoides grateloupii* (d'Orbigny, 1839)
*Oolina acuticosta* (Reuss, 1862) = *Homalohedra acuticosta* (Reuss, 1862)
*Oolina* sp. d'Orbigny, 1839
*Oolina squamosa* (Montagu, 1803) = *Favulina squamosa* (Montagu, 1803)
*Oridorsalis* sp. Andersen, 1961
*Oridorsalis umbonatus* (Reuss, 1851)
*Psammosphaera fusca* Schulze, 1875
*Pullenia bulloides* (d'Orbigny, 1846)
*Pullenia quinqueloba* (Reuss, 1851)
*Pullenia* sp. Parker & Jones, 1862
*Pyrgo murrhina* (Schwager, 1866)
*Pyrgo* sp. Defrance, 1824
*Pyrulina* sp. d'Orbigny, 1839
*Quinqueloculina arctica* Cushman, 1933
*Quinqueloculina seminula* (Linnaeus, 1758)
*Quinqueloculina* sp. d'Orbigny, 1826
*Recurvoidatus* sp. Saidova, 1970
*Reophax bradyi* Brönnimann & Whittaker, 1980
*Reophax dentaliniformis* (Brady, 1881) = *Nodulina dentaliniformis* (Brady, 1881)
*Reophax fusiformis* (Williamson, 1858)
*Robertinoides bradyi* (Cushman & Parker, 1936)
*Robertinoides* sp. Höglund, 1947
*Rosalina bradyi* (Cushman, 1915)
*Rosalina* sp. d'Orbigny, 1826
*Rosalina vilardeboana* d'Orbigny, 1839 = *Discorbis vilardeboanus* (d'Orbigny, 1839)
*Saccammina sphaerica* Brady, 1871
*Sigmoilopsis schlumbergeri* (Silvestri, 1904)
*Spiroplectinella wrightii* (Silvestri, 1903)
*Textularia* sp. Defrance, 1824
*Trifarina angulosa* (Williamson, 1858)
*Trifarina fluens* (Todd in Cushman & McCulloch, 1948)
*Trifarina* sp. Cushman, 1923
*Triloculina elongata* d'Orbigny in Fornasini, 1905
*Triloculina* sp. d'Orbigny, 1826
*Triloculina trihedra* Loeblich & Tappan, 1953
*Trochammina inflata* (Montagu, 1808)
*Uvigerina aculeata* d'Orbigny, 1846
*Uvigerina mediterranea* Hofker, 1932
*Uvigerina peregrina* Cushman, 1923
*Uvigerina* sp. d'Orbigny, 1826
- microphotograph tables of benthic foraminifera for each station and taxonomic descriptions to them (Phototable 1, Phototable 2, Phototable 3, Phototable 4, Phototable 5, Phototable 6, Phototable 7, Phototable 8, Phototable 9, Phototable 10, Phototable 11, Phototable 12, Phototable 13, Phototable 14, Phototable 15, Phototable 16, Phototable 17, Phototable 18, Phototable 19, Phototable 20, Phototable 21, Phototable 22, Phototable 23, Phototable 24, Phototable 25, Phototable 26).

**Phototable 1:**
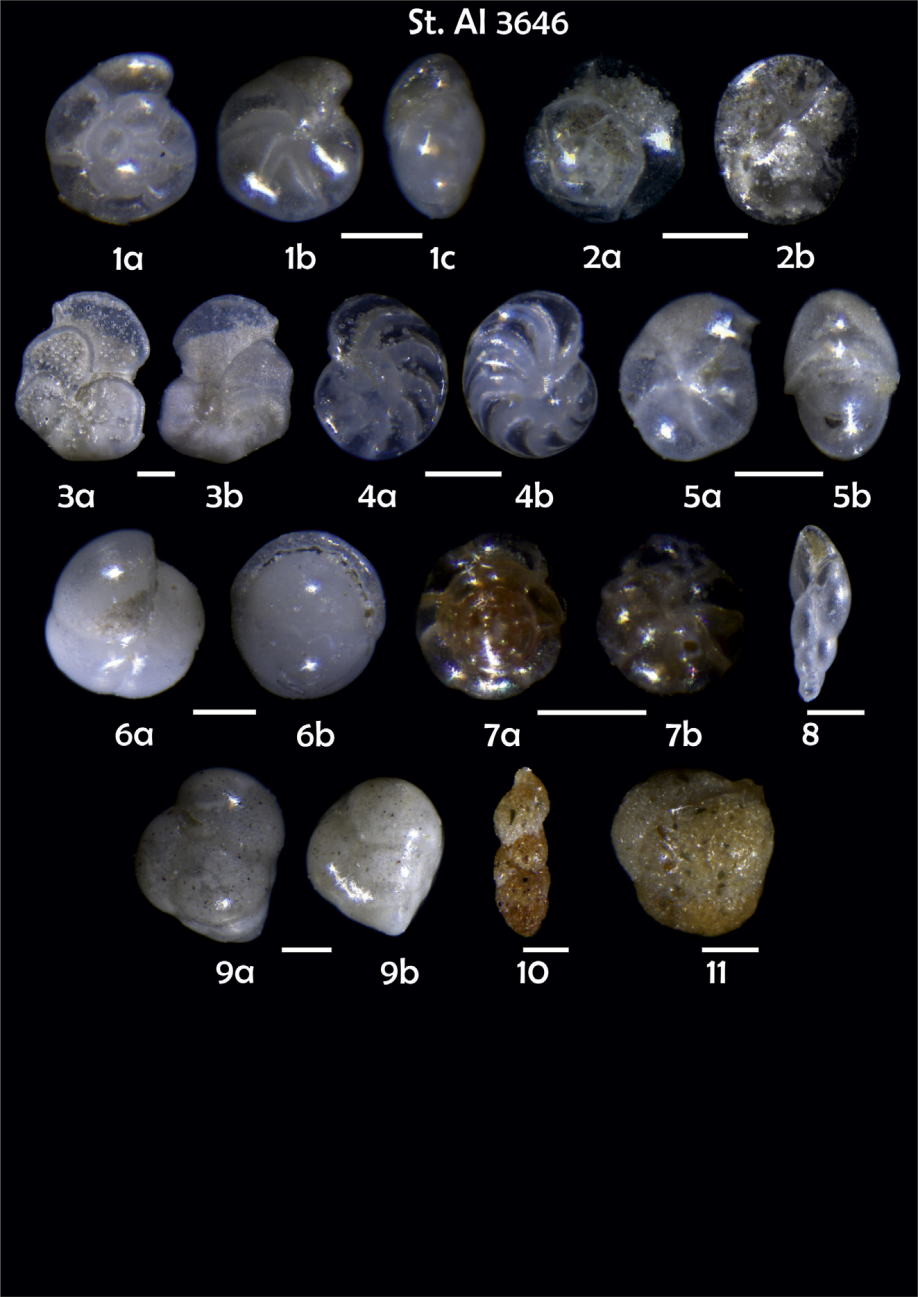
**Station 3646** **1***Oridorsalis umbonatus*, **a** spiral view, **b** umbilical view, **c** apertural view. **2***Epistominella exigua*, **a** spiral view, **b** umbilical view. **3***Cibicidoides* sp., **a** umbilical view, **b** spiral view. **4***C. wuellerstorfi*, **a** umbilical view, **b** spiral view. **5***Pullenia quinqueloba*, **a** side view, **b** apertural view. **6***P. bulloides*, **a** side view, **b** apertural view. **7***Ionella tumidula*, **a** spiral view, **b** umbilical view. **8***Fursenkoina complanata*. **9***Eggerella bradyi*, **a** apertural view, **b** lateral view. **10***Karrerulina conversa*. **11***Cribrostomoides subglobosus*. Scale 100 μm.

**Phototable 2:**
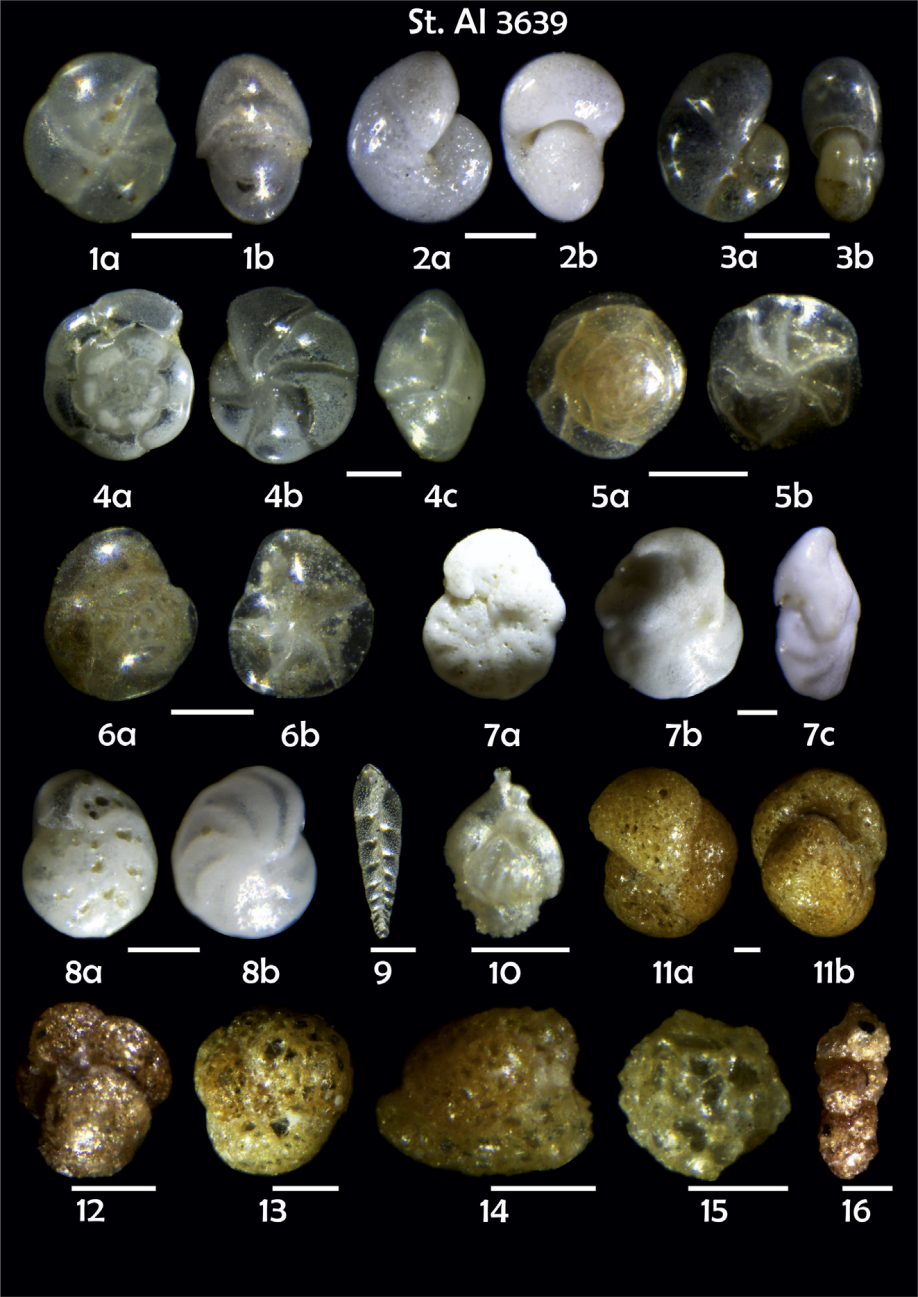
**Station 3639** **1***Pullenia quinqueloba*, **a** side view, **b** apertural view. **2***Melonis pompilioides*, **a** side view, **b** apertural view. **3***Pullenia* sp., **a** umbilical view, **b** apertural view. **4***Oridorsalis umbonatus*, **a** spiral view, **b** lateral view, **c** apertural view. **5***Alabaminella weddellensis*, **a** spiral view, **b** umbilical view. **6***Epistominella exigua*, **a** spiral view, **b** umbilical view. **7***Cibicidoides pseudoungeriana*, **a** umbilical view, **b** spiral view, **c** apertural view. **8***C. wuellerstorfi*, **a** umbilical view, **b** spiral view. **9***Bolivina earlandi.***10***Uvigerina* sp. **11***Cribrostomoides subglobosus*, **a** lateral view, **b** apertural view. **12** ?. **13** ?. **14***Adercotryma glomeratum*. **15***Psammosphaera fusca*. **16***Karrerulina conversa*. Scale 100 μm.

**Phototable 3:**
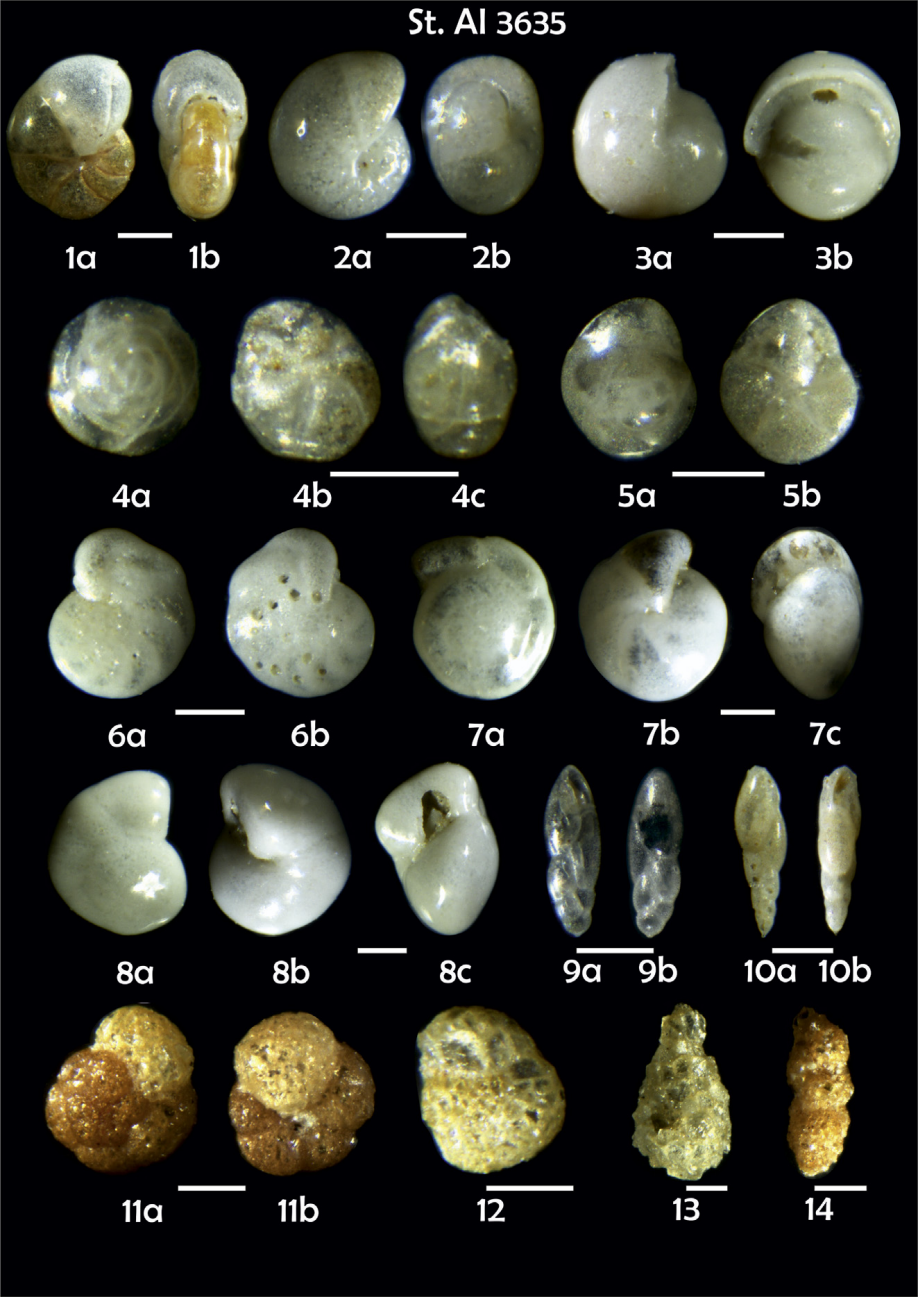
**Station 3635** **1***Pullenia* sp., **a** lateral view, **b** apertural view. **2***Melonis pompilioides*, **a** side view, **b** apertural view. **3***Pullenia bulloides*, **a** side view, **b** apertural view. **4***Alabaminella weddellensis*, **a** spiral view, **b** umbilical view, **c** lateral view. **5***Epistominella exigua*, **a** spiral view, **b** umbilical view. **6***Cibicidoides wuellerstorfi*, **a** umbilical view, **b** spiral view. **7***Oridorsalis umbonatus*, **a** spiral view, **b** umbilical view, **c** apertural view. **8***Hansenisca*? sp., **a** spiral view, **b** umbilical view, **c** apertural view. **9, 10***Fursenkoina complanata*, **9a** apertural view, **9b** lateral view, **10a** lateral view, **10b** apertural view. **11***Trochammina inflata*, **a** spiral view, **b** umbilical view. **12** ?. **13***Lagenammina difflugiformis*. **14***Karrerulina conversa*. Scale 100 μm.

**Phototable 4:**
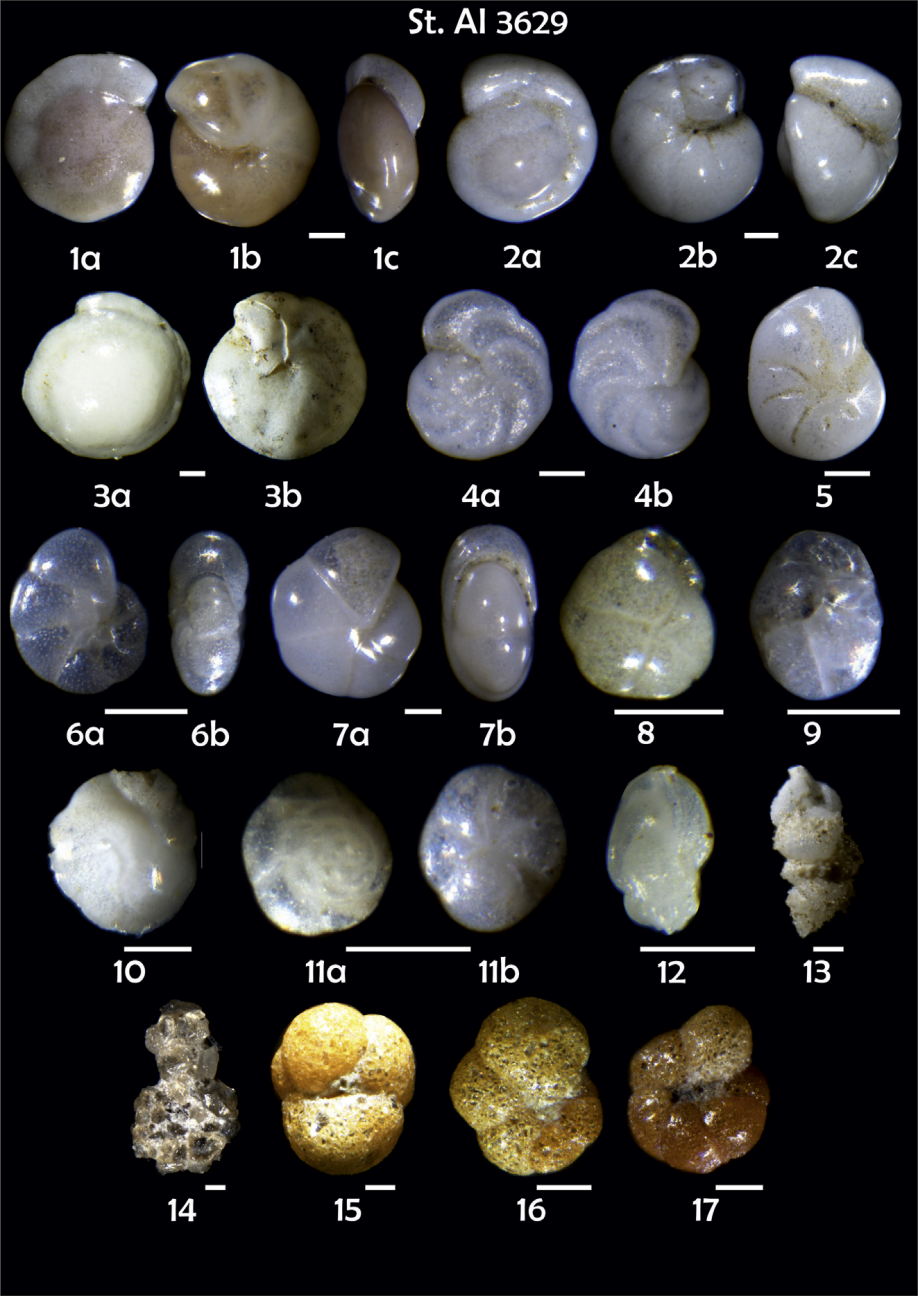
**Station 3629** **1***Gyroidina orbicularis*, **a** spiral view, **b** umbilical view, **c** apertural view. **2***Hansenisca soldanii,***a** spiral view, **b** umbilical view, **c** apertural view. **3***Oridorsalis* sp., **a** spiral view, **b** umbilical view. **4***Cibicidoides wuellerstorfi*, **a** umbilical view, **b** spiral view. **5***Elphidium incertum*?. **6***Astrononion stelligerum*, **a** side view, **b** apertural view.**7***Pullenia quinqueloba,***a** side view, **b** apertural view. **8***Cassidulina reniforme.***9***Globocassidulina subglobosa*. **10***Cassidulina laevigata*. **11***Alabaminella weddellensis*, **a** spiral view, **b** umbilical view. **12***Trifarina angulosa*. **13***Uvigerina aculeata*. **14** ?. **15** ?. **16***Trochammina inflata*. **17** ?. Scale 100 μm.

**Phototable 5:**
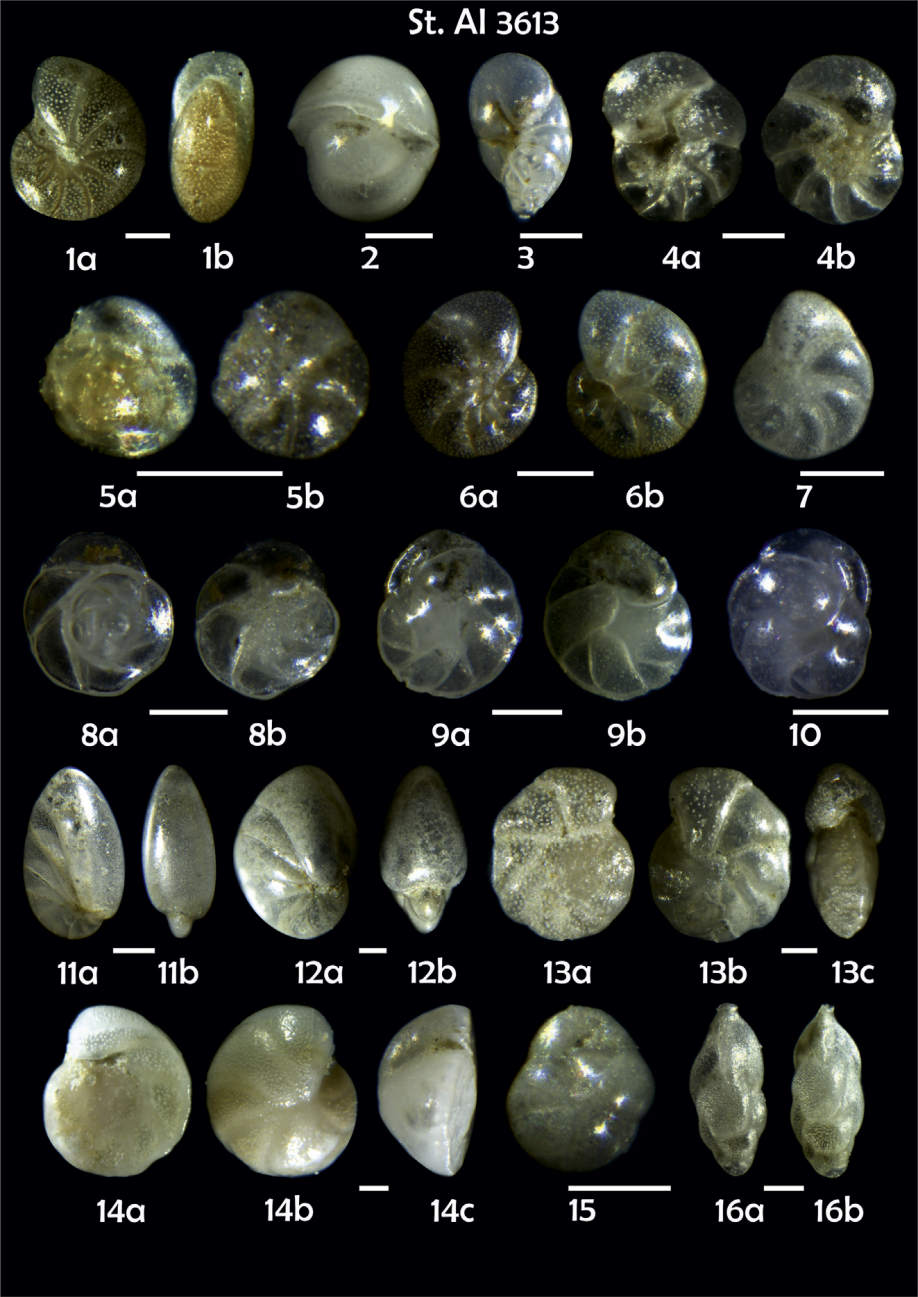

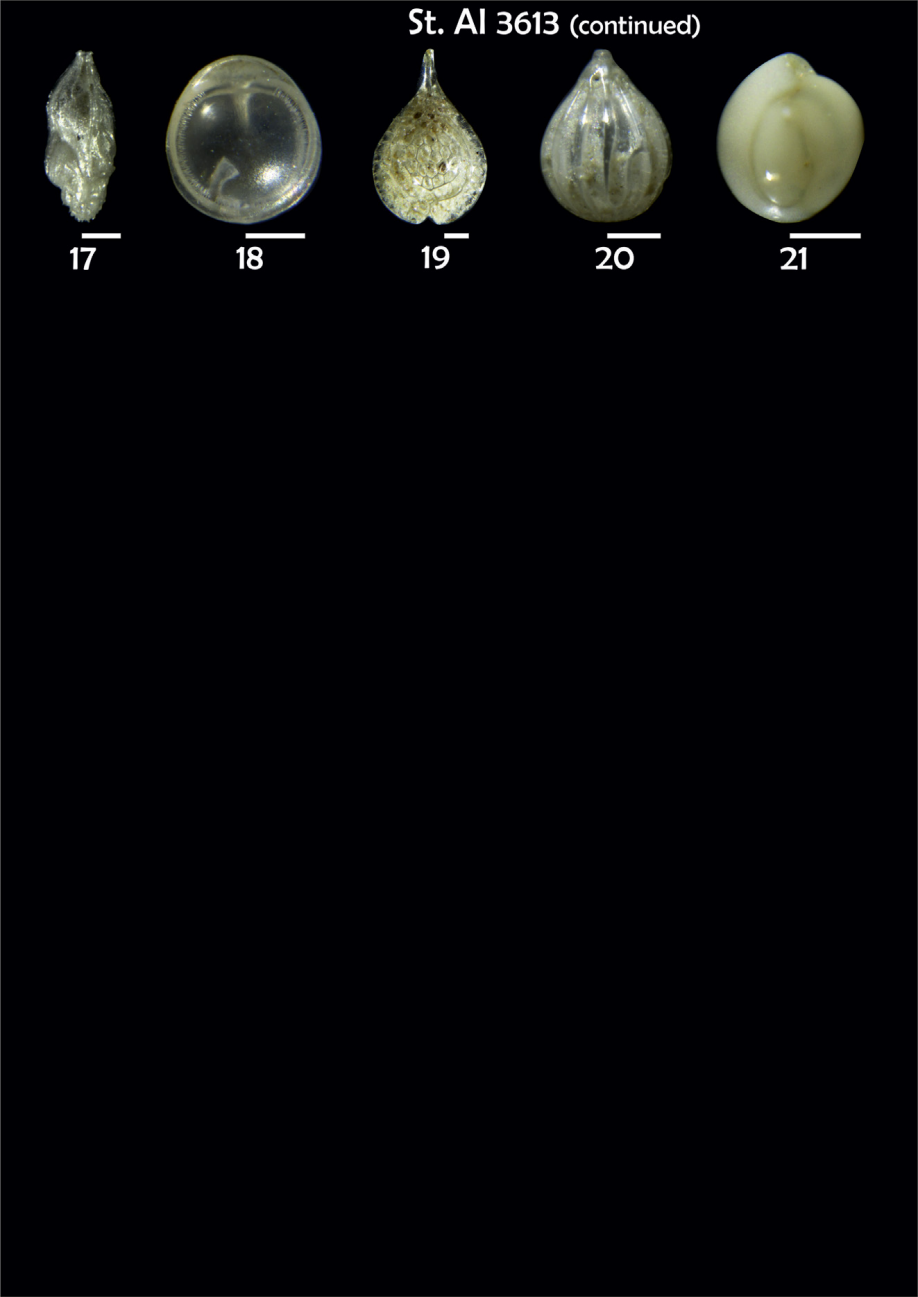
**Station 3613** **1***Melonis barleeanus,***a** side view, **b** apertural view. **2***Pullenia bulloides*. **3***Robertinoides bradyi.***4***Rosalina* sp. cf. *R. vilardeboana*?, **a** umbilical view, **b** spiral view. **5***Alabaminella weddellensis*, **a** spiral view, **b** umbilical view. **6***Astrononion stelligerum*, **a, b** side view. **7***Elphidium subarcticum*. **8***Gavelinopsis praegeri*, **a** spiral view, **b** umbilical view.**9***Cassidulina teretis*, **a** apertural view, **b** lateral view. **10***C. reniforme*. **11***Nonionella turgida*, **a** lateral view, **b** apertural view. **12***Nonionella* sp.?. **13***Cibicidoides* sp. cf. *C*. *pseudoungeriana*?, **a** umbilical view, **b** spiral view, **c** apertural view. **14***Cibicides refulgens,***a** umbilical view, **b** spiral view, **c** apertural view. **15***Globocassidulina subglobosa.***16***Trifarina fluens,***a**, **b** lateral view. **17***T. angulosa*, **a**, **b** lateral view. **18***Fissurina* sp. **19***F. squamosoalata*. **20***Oolina acuticosta*. **21***Quinqueloculina seminula*. Scale 100 μm.

**Phototable 6:**
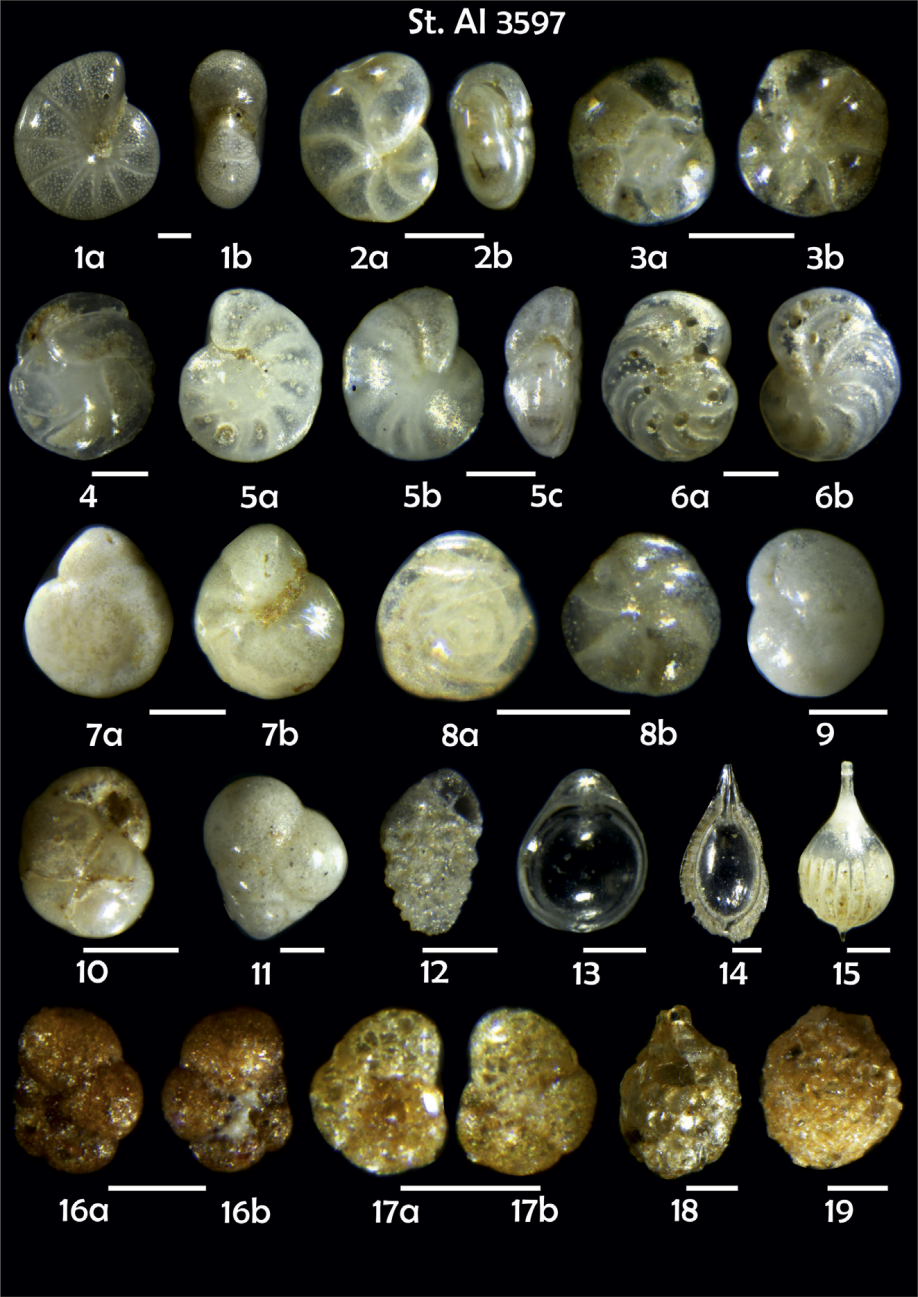
**Station 3597** **1***Melonis barleeanus,***a** side view, **b** apertural view. **2***Pullenia* sp., **a** side view, **b** apertural view. **3***Epistominella exigua*, **a** spiral view, **b** umbilical view. **4***Cassidulina teretis*. **5***Cibicidoides wuellerstorfi*, **a** umbilical view, **b** spiral view, **c** apertural view. **6***C. wuellerstorfi*, **a** umbilical view, **b** spiral view. **7***Hanseniska* sp.?, **a** spiral view, **b** umbilical view. **8***Alabaminella weddellensis*, **a** spiral view, **b** umbilical view. **9***Cassidulina reniforme*. **10***Globacassidulina subglobosa*. **11***Eggerella bradyi*. **12***Bolivina pseudoplicata*. **13**, **14***Fissurina* spp. **15***Lagena* sp. **16** ?, **a, b** side view. **17***Trochammina inflata*, **a** spiral view, **b** umbilical view. **18***Saccammina sphaerica*. **19***Psammosphaera fusca.* Scale 100 μm.

**Phototable 7:**
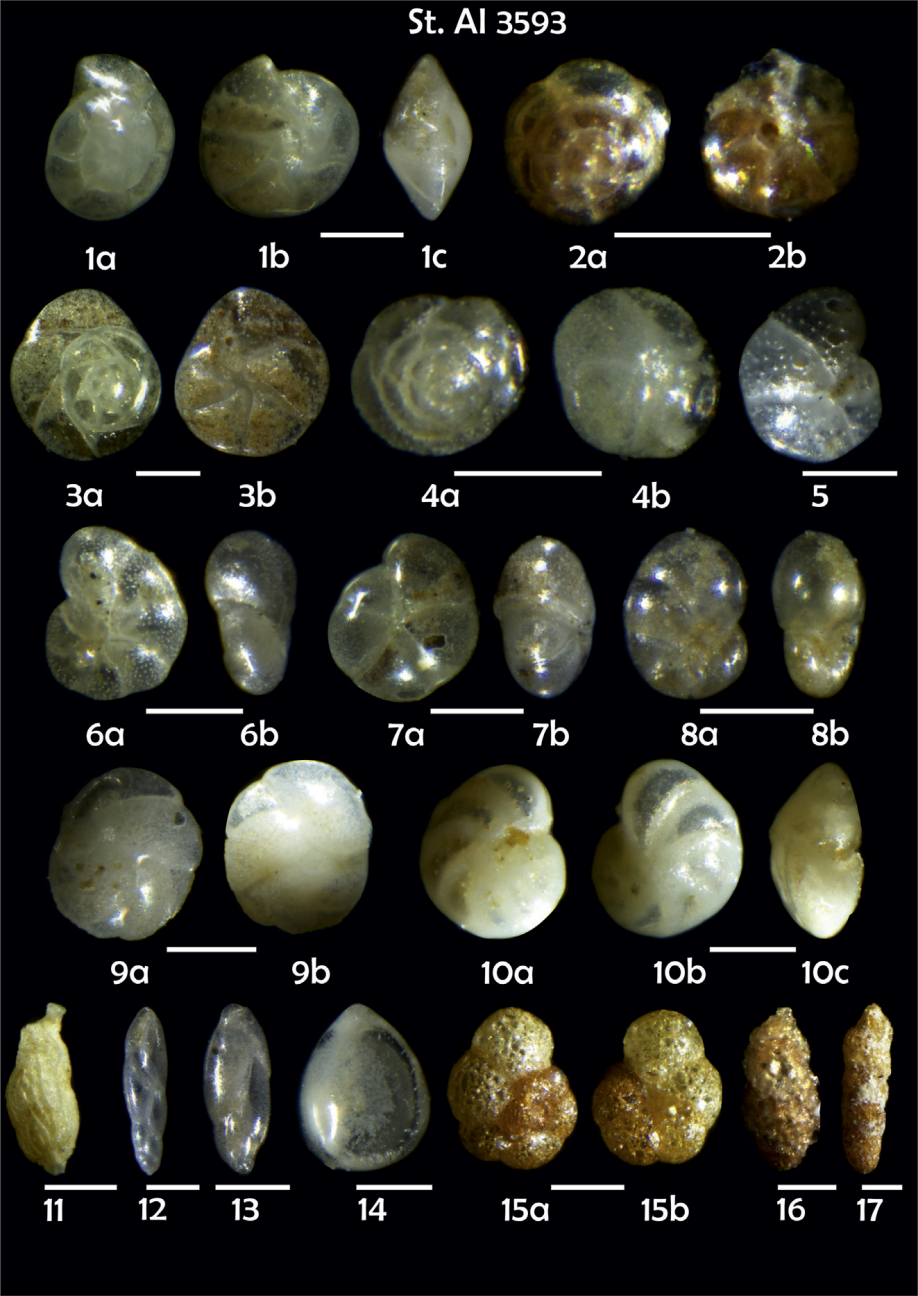
**Station 3593** **1***Oridorsalis umbonatus*, **a** spiral view, **b** umbilical view, **c** apertural view. **2***Ioanella tumidula,***a** spiral view, **b** umbilical view. **3***Epistominella exigua***, a** spiral view, **b** umbilical view. **4***Alabaminella weddellensis*, **a** spiral view, **b** umbilical view. **5***Melonis* sp. juvenile form? like st.3592 (9). **6***Astrononion stelligerum*, **a** side view, **b** apertural view. **7***Pullenia quinqueloba*, **a** side view, **b** apertural view. **8***Nonionella auricula*, **a** lateral view, **b** apertural view. **9***Cassidulina laevigata*, **a** apertural view, **b** lateral view. **10***Cibicidoides wuellerstorfi*, **a** umbilical view, **b** spiral view, **c** apertural view. **11***Trifarina angulosa.***12***Fursenkoina complanata*. **13***F. fusiformis*. **14***Fissurina* sp. **15***Trochammina inflata*. **16***Karrerulina* sp. **17***Karrerulina conversa*. Scale 100 μm.

**Phototable 8:**
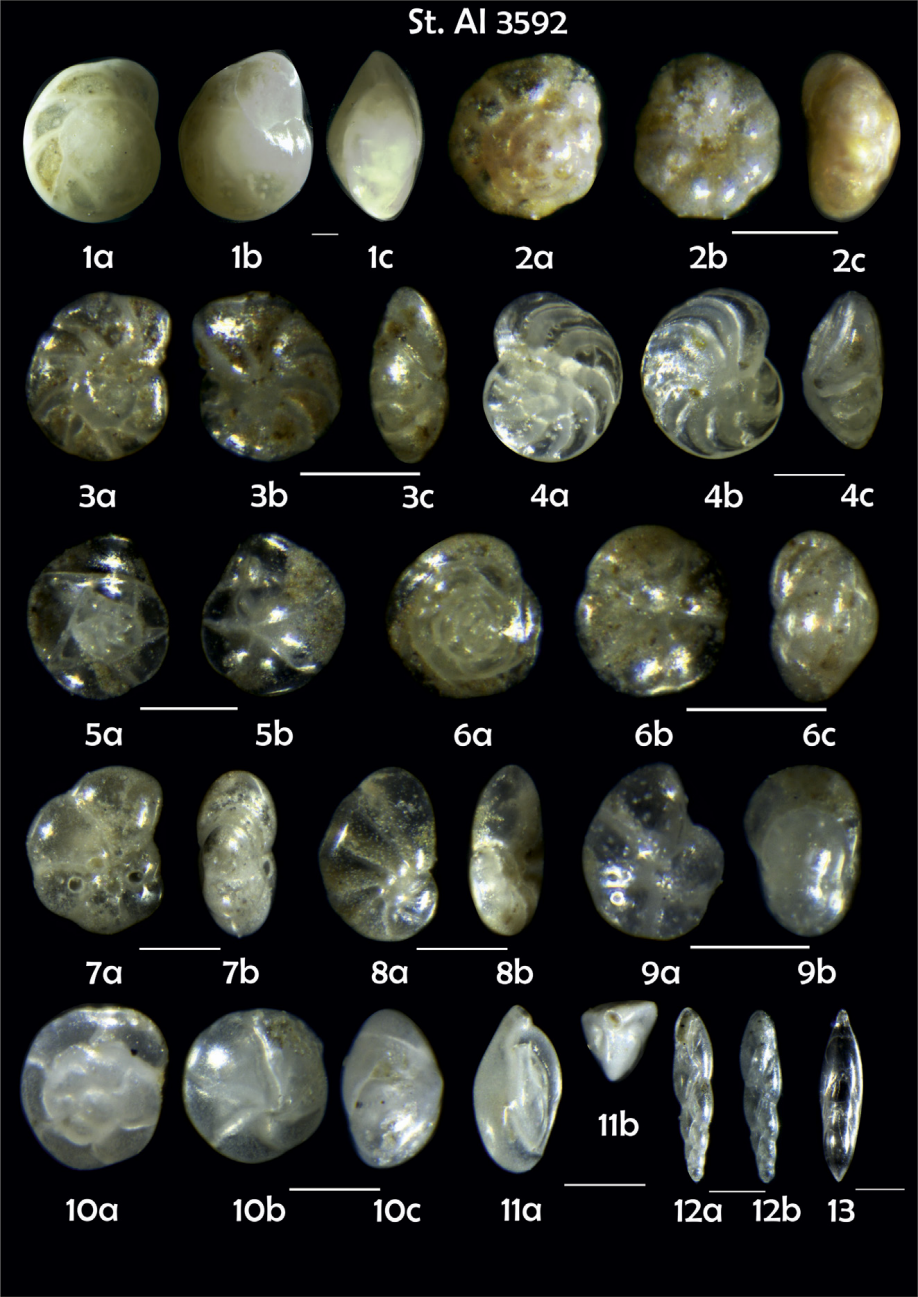
**Station 3592** **1***Hoeglundina elegans*, **a** spiral view, **b** umbilical view, **c** lateral view. **2**. *Ioanella tumidula*, **a** spiral view, **b** umbilical view, **c** lateral view. **3***Cibicidoides* sp. **4***C. wuellerstorfi,***a** umbilical view, **b** spiral view, **c** apertural view. **5***Epistominella exigua*, **a** spiral view, **b** umbilical view. **6***Alabaminella weddellensis*, **a** spiral view, **b** umbilical view, **c** lateral view. **7***Pullenia quinqueloba,***a** side view, **b** apertural view. **8**. *Nonionella auricula*, **a** spiral view, **b** apertural view **9**. *Melonis* sp. juvenile form?, lake st. 3593 (5), **a** side view, **b** apertural view. **10***Oridorsalis umbonatus*, **a** spiral view, **b** umbilical view, **c** lateral view. **11***Triloculina trihedra*, **a** lateral view, **b** apertural view. **12***Fursenkoina complanata*, **a** apertural view, **b** lateral view. **13***Pyrulina* sp. Scale 100 μm.

**Phototable 9:**
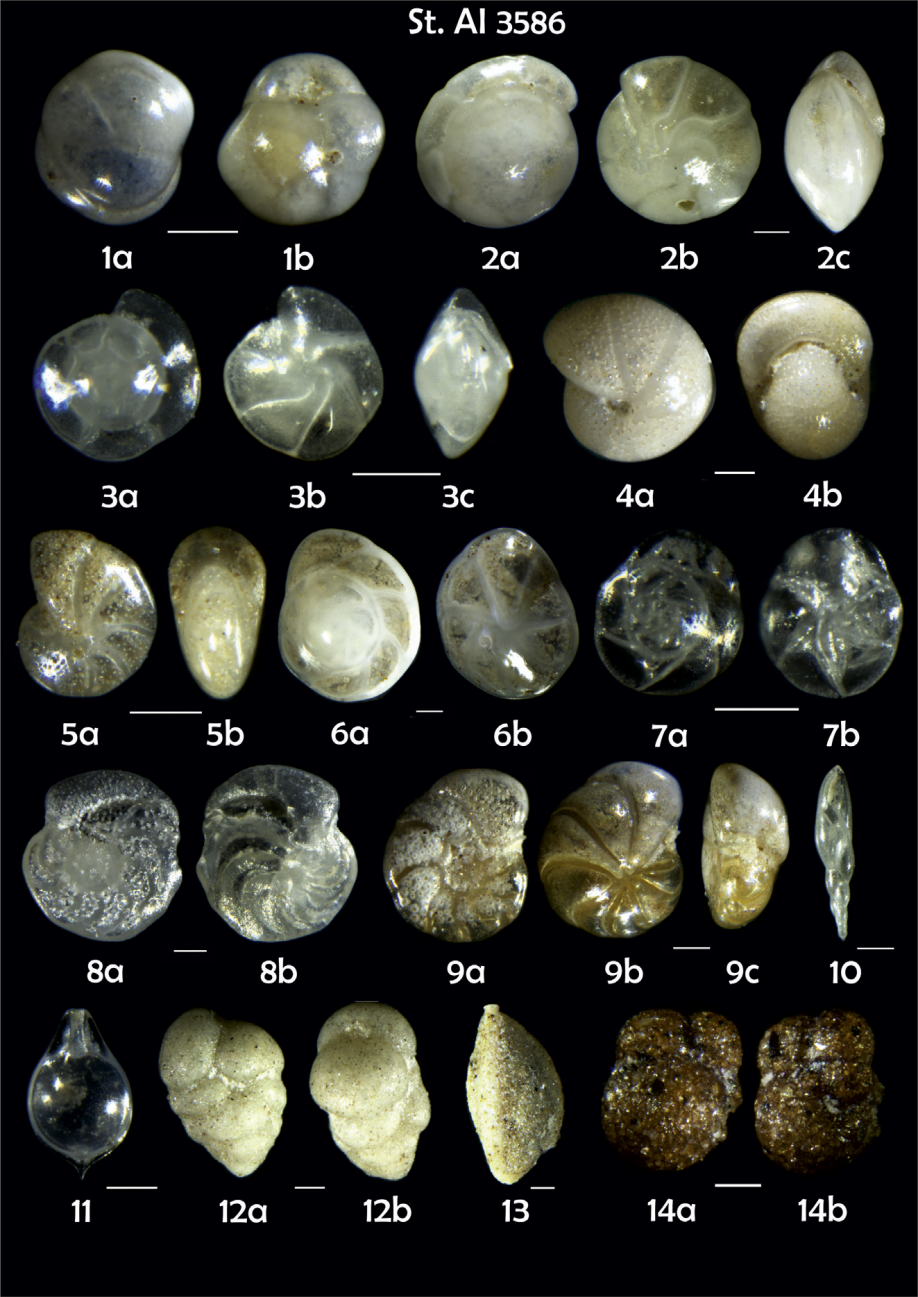
**Station 3586** **1***Globocaccidulina subglobosa*, **a** lateral view, **b** apertural view. **2, 3***Oridorsalis umbonatus*, **a** spiral view, **b** umbilical view, **c** apertural view. **4***Melonis pompilioides*, **a** side view, **b** apertural view. **5***M. barleeanus,***a** side view, **b** apertural view. **6***Hoeglundina elegans*, **a** spiral view, **b** umbilical view. **7***Epistominella exigua*, **a** spiral view, **b** umbilical view. **8***Cibicidoides wuellerstorfi,***a** umbilical view, **b** spiral view. **9***C. lobatulus*, **a** umbilical view, **b** spiral view, **c** apertural view. **10***Fursenkoina complanata*. **11***Fissurina incomposita*, **12***Karreriella bradyi*, **a** lateral view, **b** apertural view. **13***Sigmoilopsis* sp. **14** ?. Scale 100 μm.

**Phototable 10:**
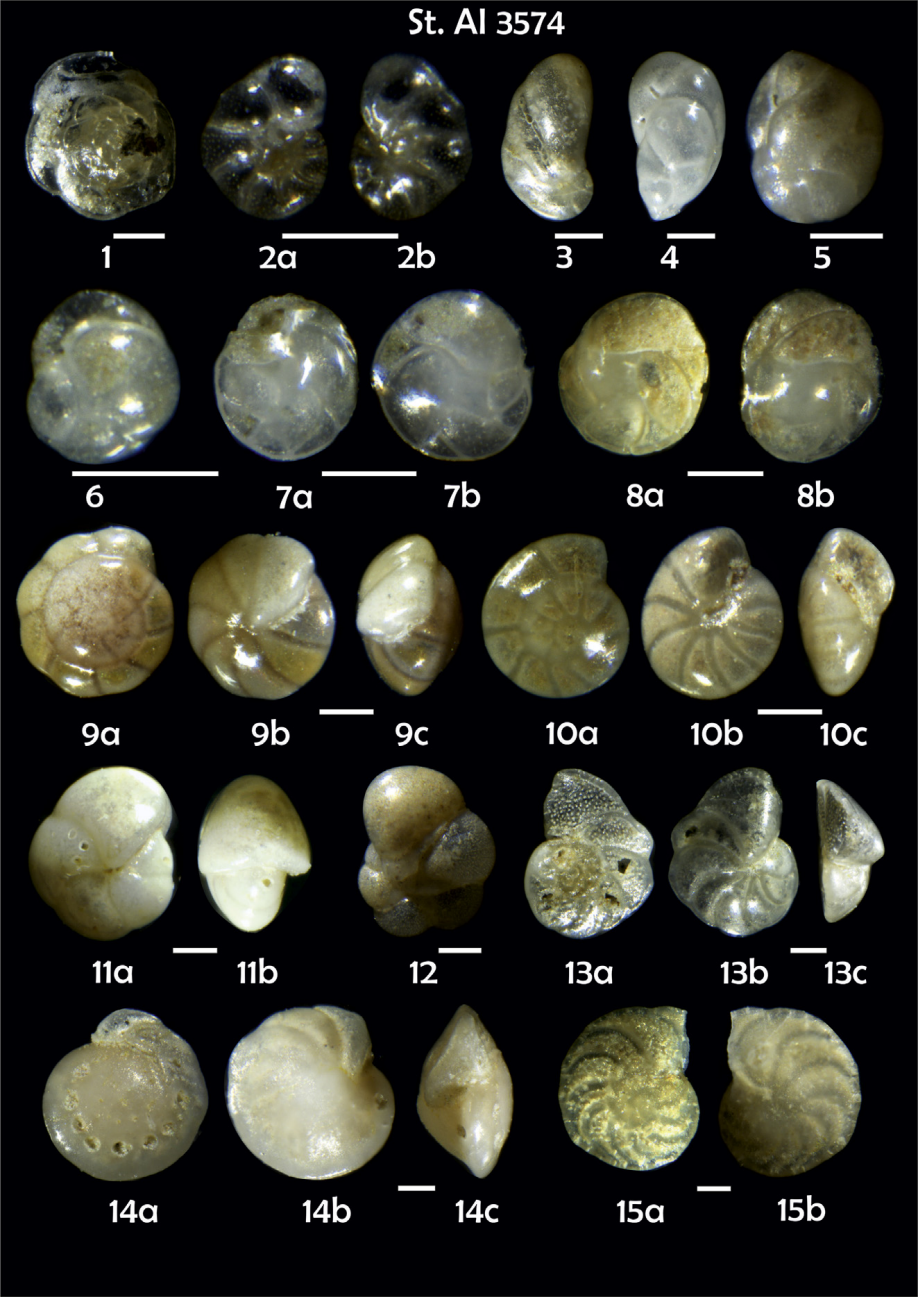

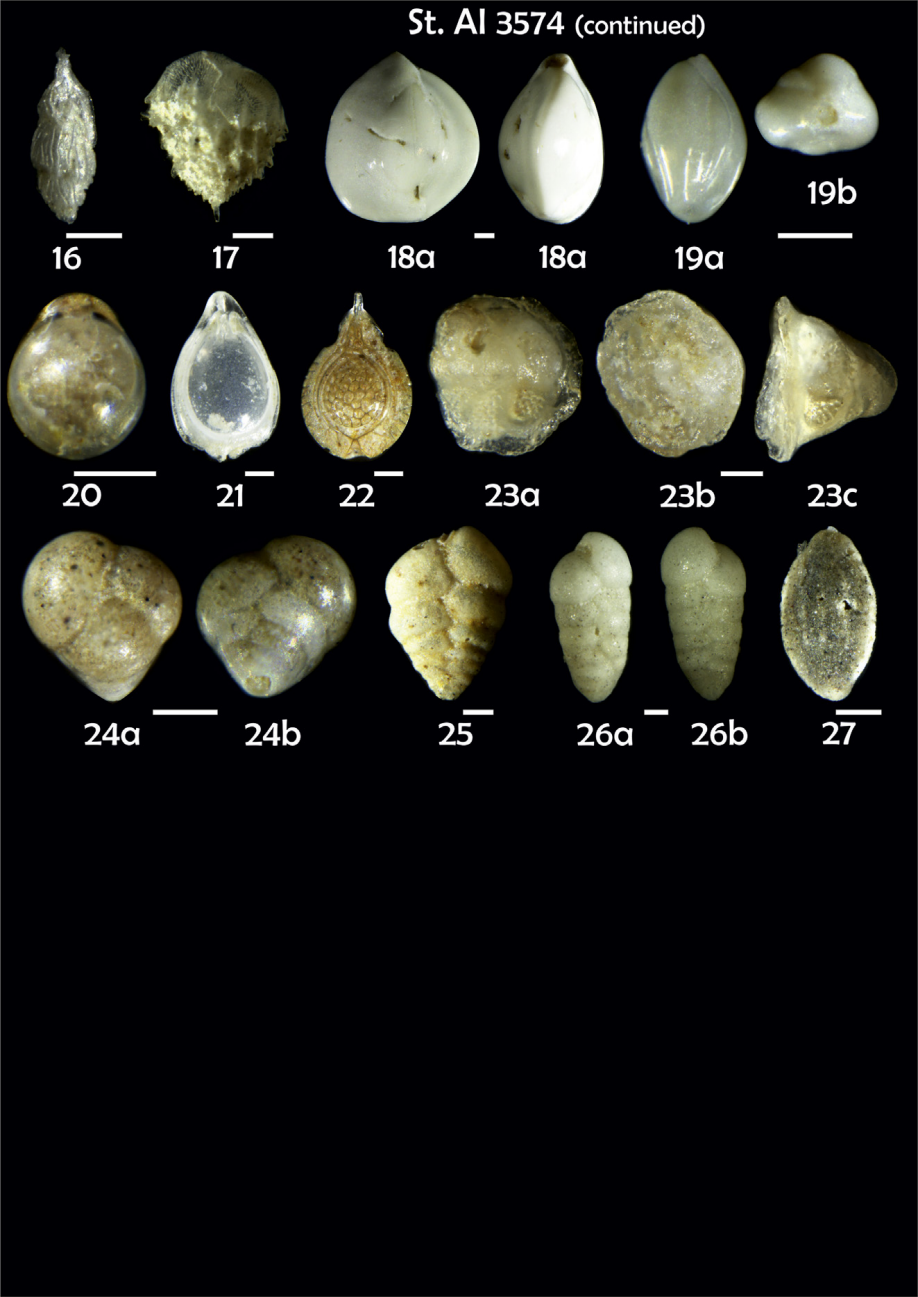
**Station 3574** **1** Gavelinopsis praegeri. **2** Nonionella auricula, **a** spiral view, **b** umbilical view. **3** Cassidulinoides bradyi. **4** Robertinoides bradyi. **5** Globocassidulina subglobosa. **6** Cassidulina reniforme. **7** C. teretis, **a** apertural view, **b** lateral view. **8** C. teretis, **a**, **b** lateral view. **9**, **10** Gyroidina orbicularis, **a** spiral view, **b** umbilical view, **c** apertural view. **11** Pullenia quinqueloba, **a** side view, **b** apertural view. **12** Astrononion gallowayi. **13** Cibicidoides lobatulus, **a** umbilical view, **b** spiral view, **c** apertural view. **14** C. pachyderma, **a** umbilical view, **b** spiral view, **c** apertural view. **15** C. wuellerstorfi?, **a** umbilical view, **b** spiral view. **16** Trifarina angulosa. **17** Bulimina mexicana. **18** Triloculina sp. **19** Quinqueloculina sp., **a** lateral view, **b** apertural view. **20**, **21** Fissurina spp. **22** F. lacunata. **23** Carpenteria balaniformis, **a, b** side view, **c** lateral view. **24** Eggerella bradyi, **a** apertural view, **b** lateral view. **25** Textularia sp. **26** Karreriella bradyi, **a** apertural view, **b** lateral view. **27** Sigmoilopsis schlumbergeri. Scale 100 μm.

**Phototable 11:**
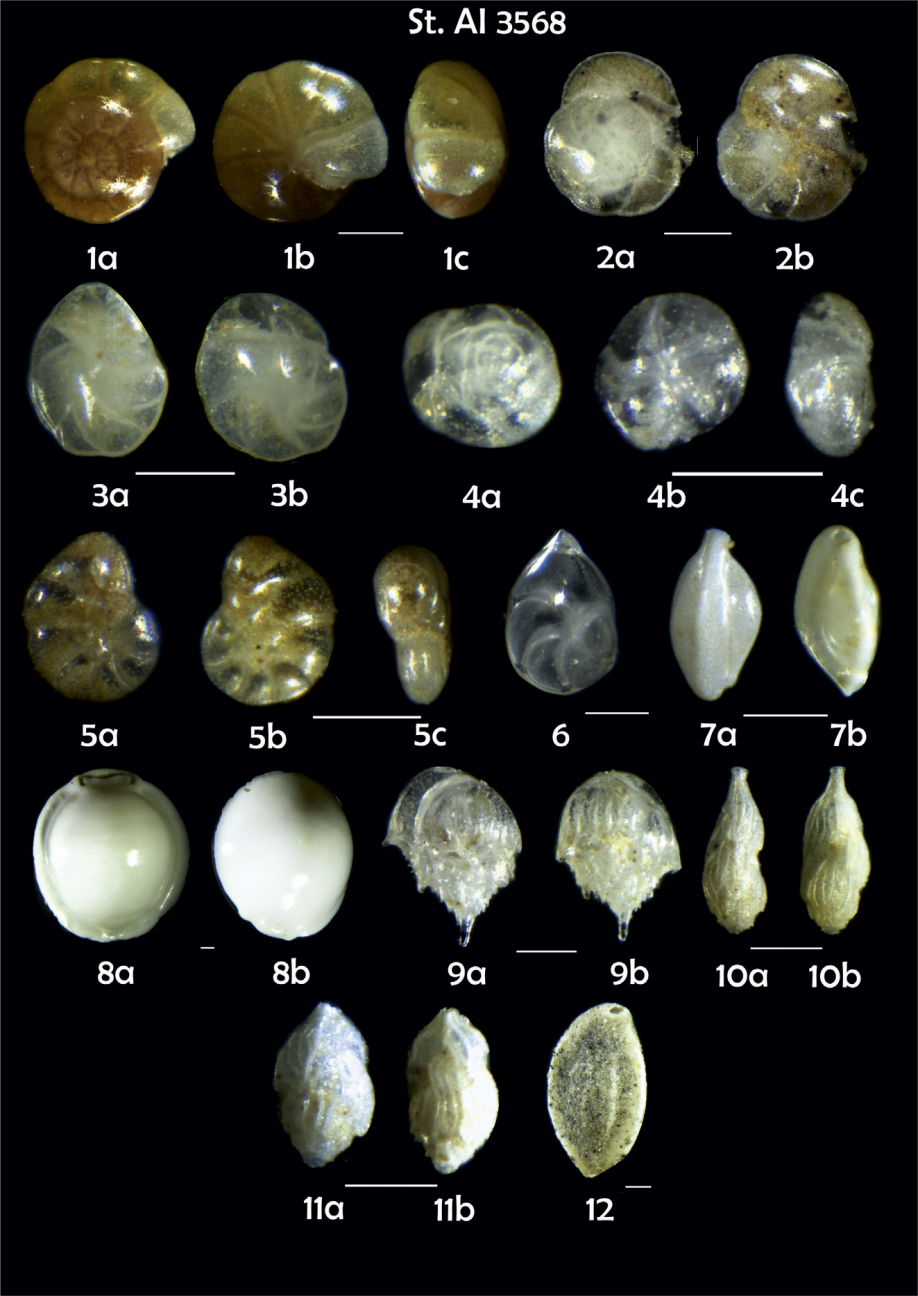
**Station 3568** **1***Gyroidina orbicularis*, **a** spiral view, **b** umbilical view, **c** lateral view.**2***Gavelinopsis praegeri*, **a** spiral view, **b** umbilical view. **3***Cassidulina teretis*, **a** lateral view, **b** apertural view. **4***Alabaminella weddellensis*, **a** spiral view, **b** umbilical view, **c** lateral view. **5** ? **a**, umbilical, **b** spiral view, **c** apertural view. **6***Lenticulina gibba*. **7***Triloculina trihedra***a**, **b** lateral view. **8***Pyrgo murrhina*, **a**, **b** lateral view**. 9***Bulimina mexicana*, **a**, **b** lateral view. **10***Trifarina angulosa*, **a**, **b** side view. **11***Trifarina*? sp., **a**, **b** side view. **12***Sigmoilopsis schlumbergeri*. Scale 100 μm.

**Phototable 12:**
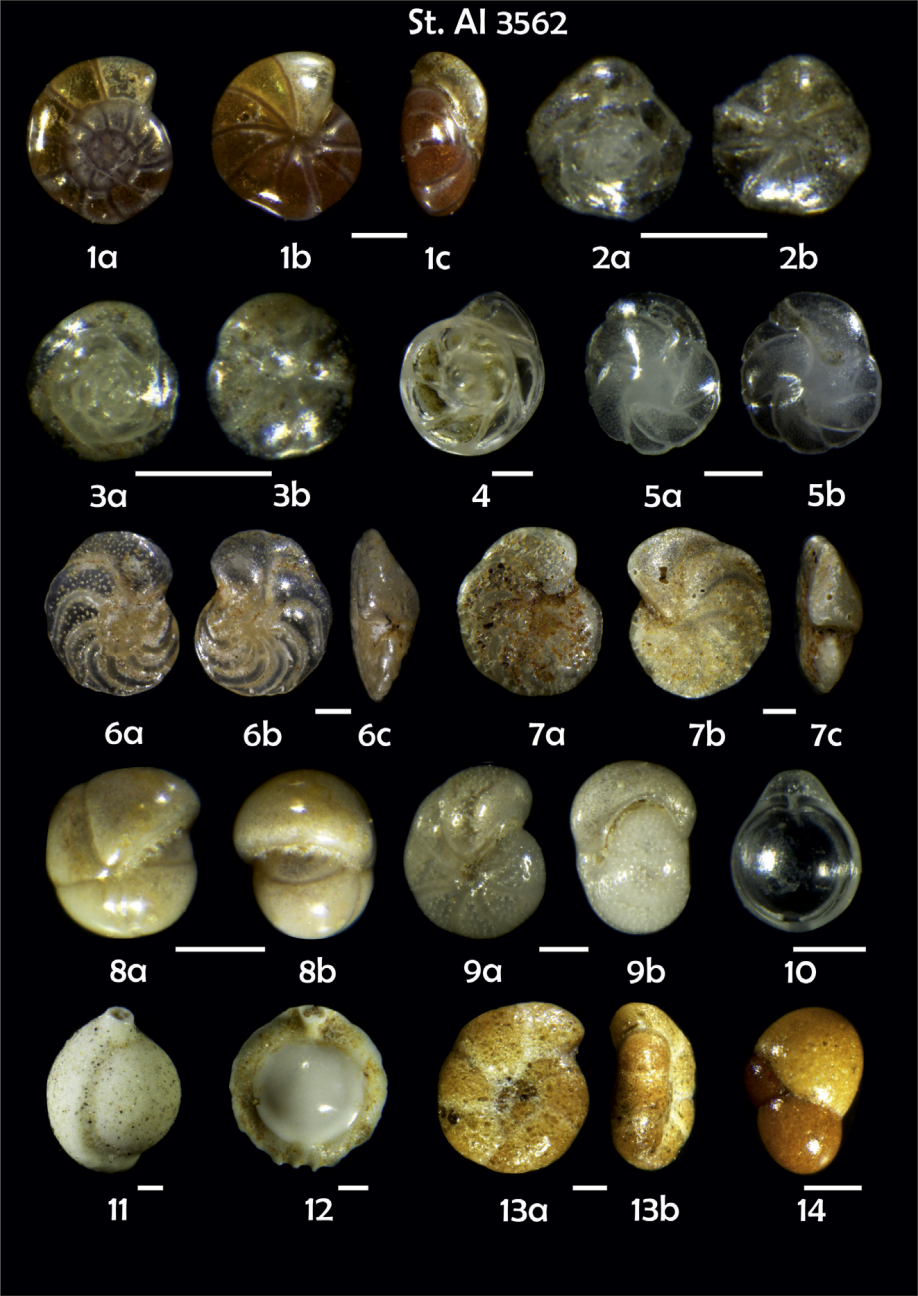
**Station 3562** **1***Gyroidina orbicularis*, **a** spiral view, **b** umbilical view, **c** apertural view. **2***Epistominella exigua*, **a** spiral view, **b** umbilical view. **3***Alabaminella weddellensis*, **a** spiral view, **b** umbilical view. **4***Hoeglundina elegans*. **5***Cassidulina teretis*, **a** apertural view, **b** lateral view. **6**, **7***Cibicidoides wuellerstorfi*, **a** umbilical view, **b** spiral view, **c** apertural view. **8***Pullenia bulloides*, **a** side view, **b** apertural view. **9***Melonis pompilioides***, a** side view, **b** apertural view. **10***Fissurina* sp. **11***Pyrgo* sp. **12***Pyrgo murrhina*. **13***Recurvoidatus*? sp., **a** umbilical view, **b** apertural view. **14***Cystammina pauciloculata*. Scale 100 μm.

**Phototable 13:**
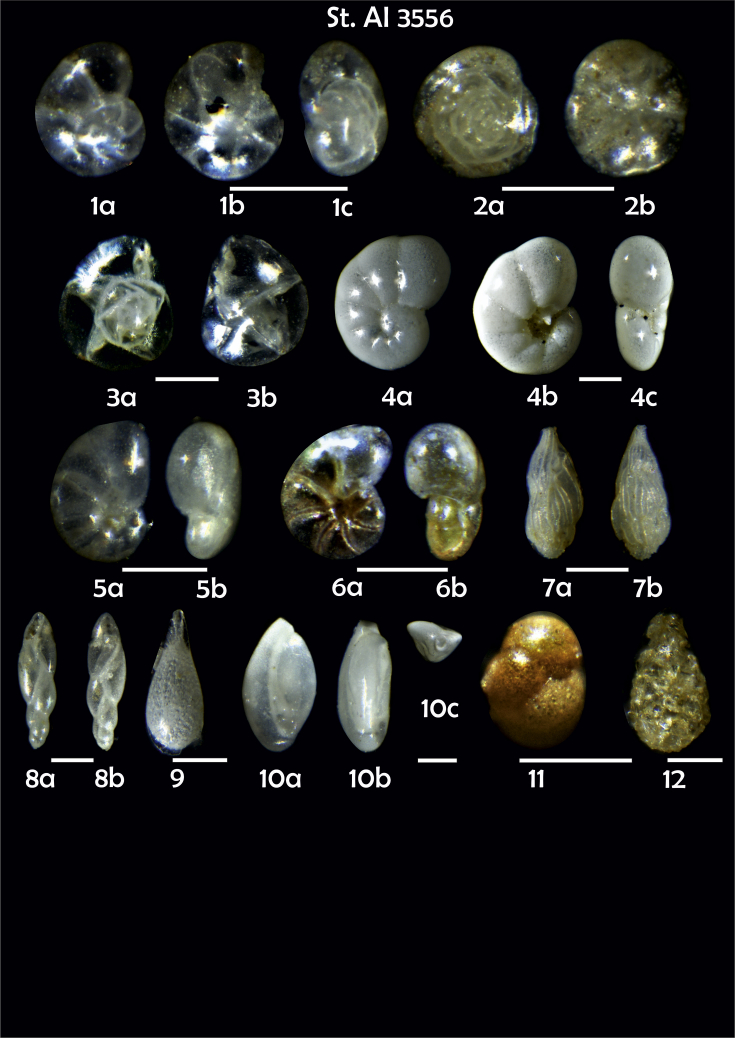
**Station 3556** **1** ?, like 3548 (4), **a** spiral view, **b** umbilical view, **c** apertural view. **2***Alabaminella weddellensis*, **a** spiral view, **b** umbilical view. **3***Epistominella exigua,***a** spiral view, **b** umbilical view. **4***Nonionoides grateloupii,***a** spiral view, **b** umbilical view, **c** apertural view. **5***Nonionella auricula*, **a** umbilical view, **b** apertural view. **6***Pullenia* sp., **a** lateral view, **b** apertural view. **7***Trifarina angulosa*, **a**, **b** lateral view. **8***Fursenkoina complanata*, **a** apertural view, **b** lateral view. **9***Lagena* sp. **10***Triloculina trihedra*, **a**, **b** lateral view, **c** apertural view. **11***Cystammina pauciloculata*. **12***Lagenammina difflugiformis*. Scale 100 μm.

**Phototable 14:**
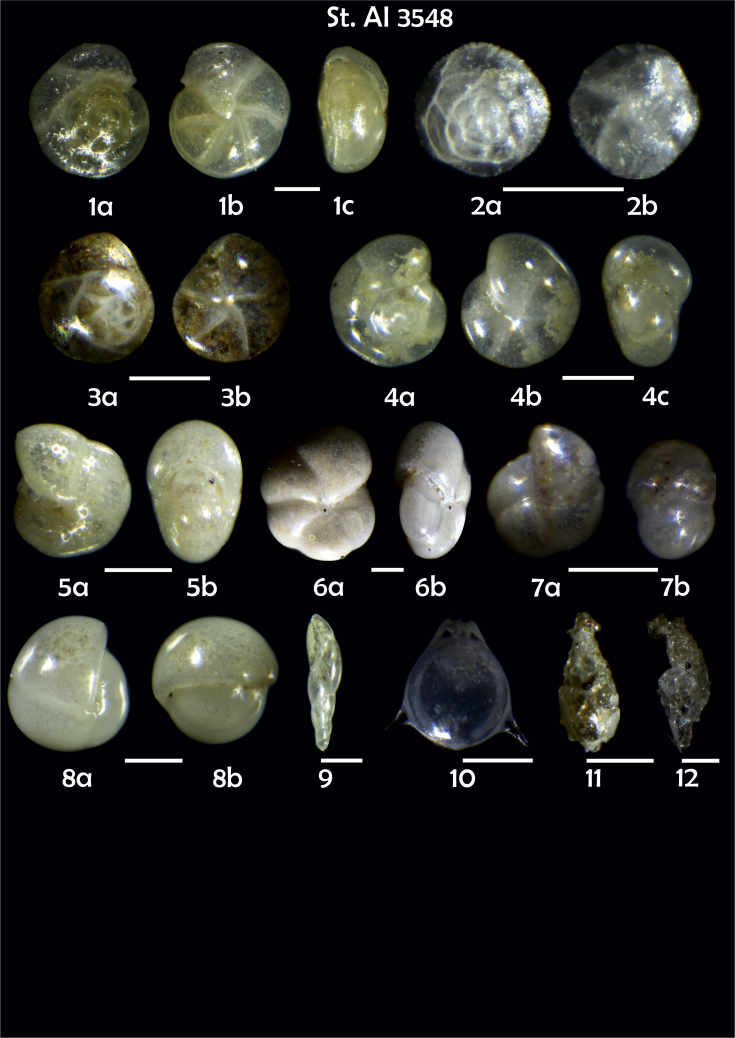
**Station 3548** **1***Cibicidoides bradyi*, **a** umbilical view, **b** spiral view, **c** apertural view. **2***Alabaminella weddellensis,***a** spiral view, **b** umbilical view. **3***Epistominella exigua,***a** spiral view, **b** umbilical view. **4** ?, like 3556 (1), **a** unbilical view, **b** spiral view, **c** apertural view. **5***Melonis* sp. cf. *M*. *barleeanus*, **a** side view, **b** apertural view. **6***Pullenia* sp., **a** side view, **b** apertutal view. **7***P. bulloides*, **a** side view, **b** apertural view. **8***P. bulloides*, **a** side view, **b** apertural view. **9***Fursenkoina complanata.****10****Fissurina staphyllearia*. **11***Lagenammina difflugiformis.***12***Reophax fusiformis*?. Scale 100 μm.

**Phototable 15:**
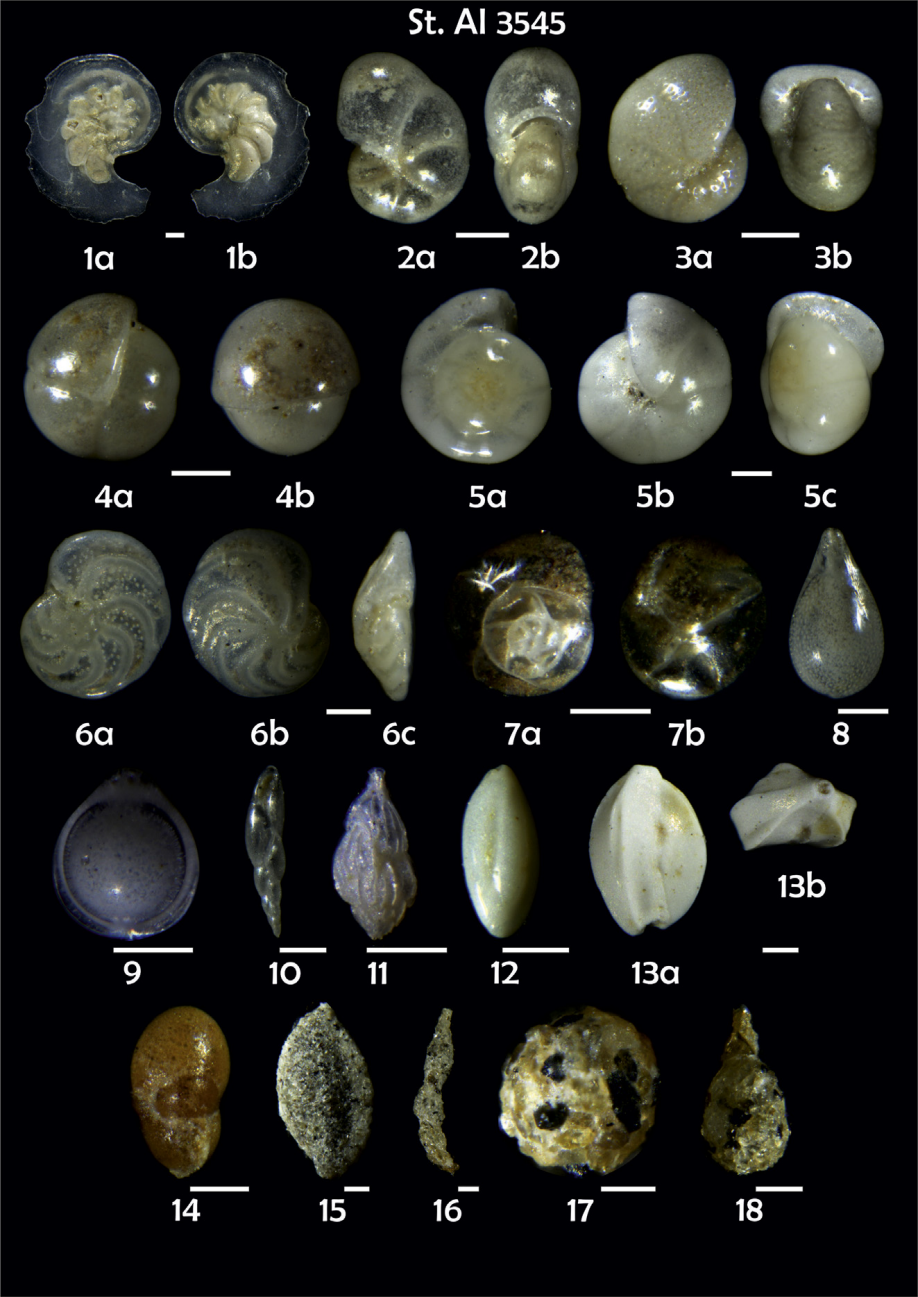
**Station 3545** ***1****Laticarinina pauperata*. **2***Pullenia* sp., **a** lateral view, **b** apertural view. **3***Melonis pompilioides*, **a** side view, **b** apertural view. **4***Pullenia bulloides*, **a** side, **b** apertural view. **5***Hansenisca soldanii,***a** spiral view, **b** umbilical view, **c** apertural view. **6***Cibicidoides wuellerstorfi,***a** umbilical view, **b** spiral view, **c** apertural view. **7***Epistominella exigua*, **a** spiral view, **b** umbilical view. **8***Lagena* sp. **9***Fissurina* sp. **10***Fursenkoina complanata*. **11***Trifarina angulosa*; **12***Triloculina elongata***13***Quinqueloculina* sp. cf. *Q. arctica*, **a** lateral view, **b** apertural view. **14***Cystammina pauciloculata*. **15***Sigmoilopsis schlumbergeri*. **16***Reophax dentaliniformis*?. **17***Psammosphaera fusca*. **18***Lagenammina difflugiformis*. Scale 100 μm.

**Phototable 16:**
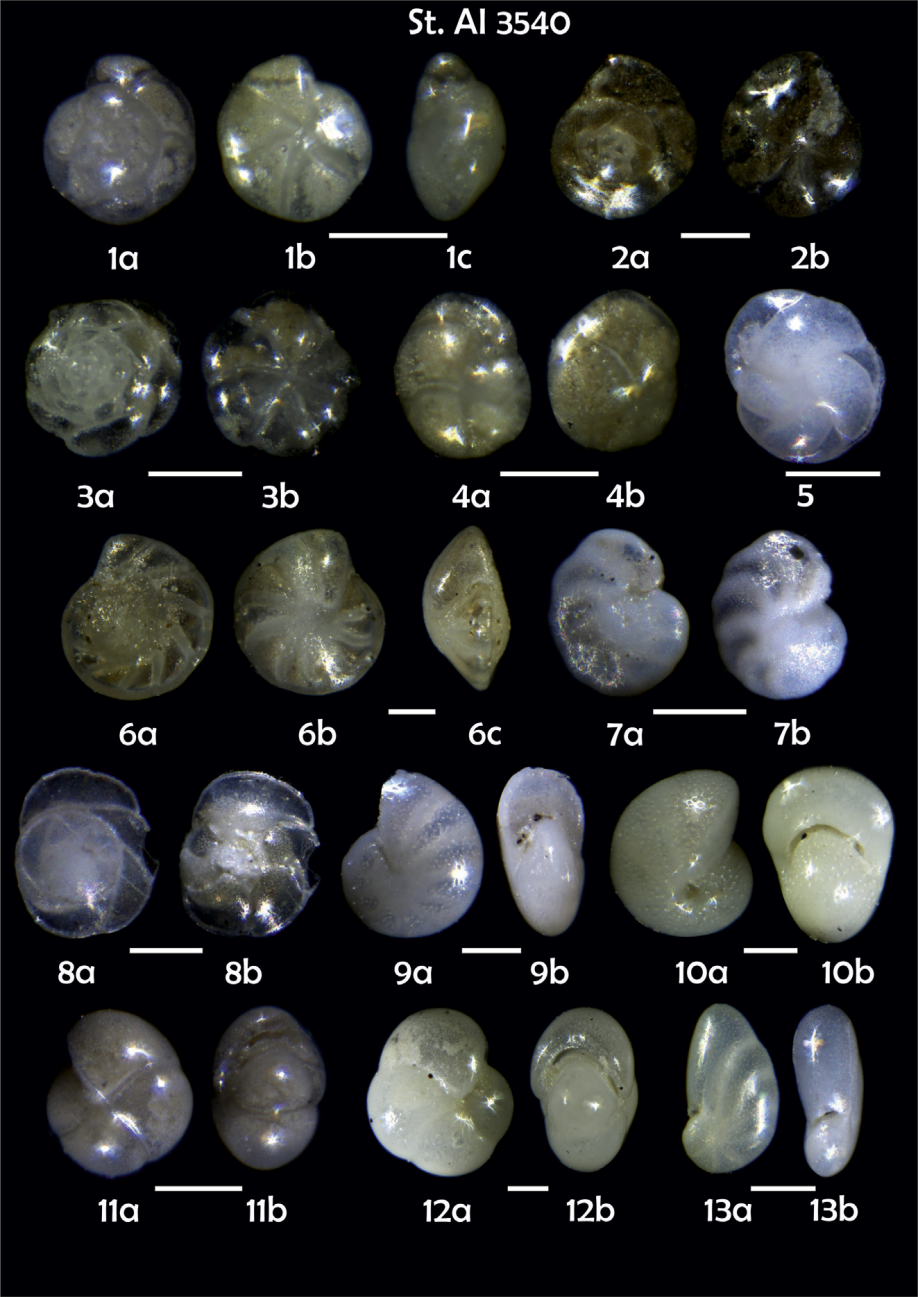

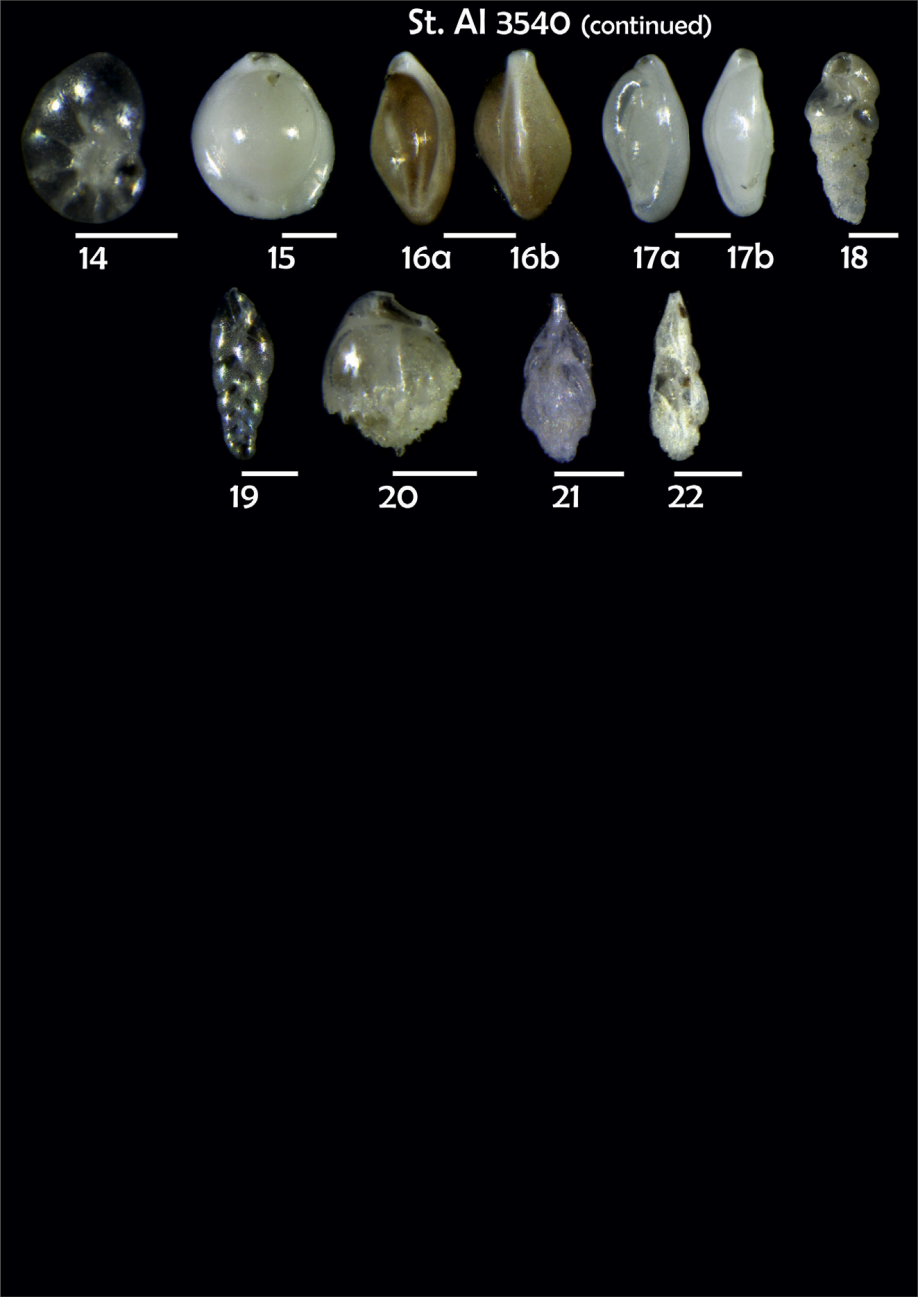
**Station 3540** **1***Oridorsalis umbonatus*, **a** spiral view, **b** umbilical view, **c** apertural view. **2***Epistominella exigua,***a** spiral view, **b** umbilical view. **3***Alabaminella weddellensis*, **a** spiral view, **b** umbilical view. **4***Cassidulina reniforme*, **a** apertural view, **b** lateral view. **5***Cassidulina laevigata*. **6***Cibicidoides* sp. cf. *C. pachyderma*?, **a** umbilical view, **b** spiral view, **c** apertural view. **7***C. lobatulus*, **a** umbilical view, **b** spiral view. **8***Gavelinopsis praegeri,***a** spiral view**, b** umbilical view. **9***Melonis barleeanus*, **a** side view, **b** apertural view. **10***Melonis pompilioides*, **a** side view, **b** apertural view. **11**, **12***Pullenia bulloides*, **a** side view, **b** apertural view. **13***Nonionella* sp. cf. *Nonionella turgida*, **a** lateral view, **b** apertural view. **14***Nonionella auricula*. **15***Pyrgo* sp. **16**, **17***Triloculina trihedra*, **a**, **b** lateral view. **18***Brizalina subspinescens*. **19***Bolivina earlandi*. **20***Bulimina mexicana*. **21, 22***Trifarina angulosa*. Scale 100 μm.

**Phototable 17:**
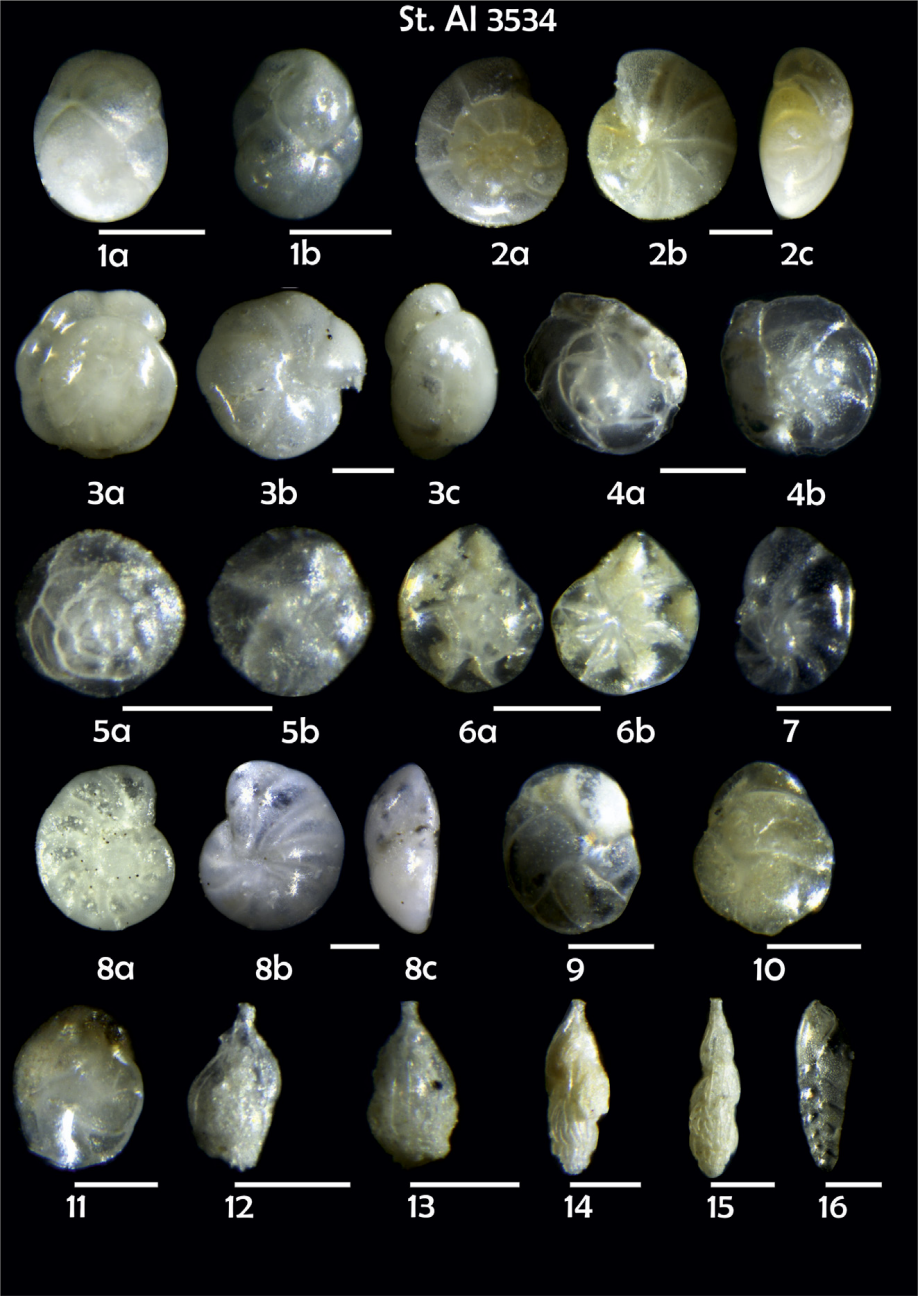
**Station 3534** **1***Globacassidulina subglobosa*, **a**, lateral view, **b** apertural view. **2***Gyroidina orbicularis*, **a** spiral view, **b** umbilical view, **c** apertural view. **3***Oridorsalis umbonatus?*, **a** spiral view, **b** umbilical view, **c** apertural view. **4***Gavelinopsis praegeri,***a** spiral view**, b** umbilical view. **5***Alabaminella weddellensis*, **a** spiral view, **b** umbilical view. **6***Epistominella exigua,***a** spiral view, **b** umbilical view. **7***Nonionella auricula*. **8***Cibicidjides lobatulus*?, **a** umbilical view, **b** spiral view. **9**, **10***Cassidulina laevigata*. **11***C. teretis*. **12, 13, 14, 15***Trifarina angulosa*. **16***Bolivina earlandi*. Scale 100 μm.

**Phototable 18:**
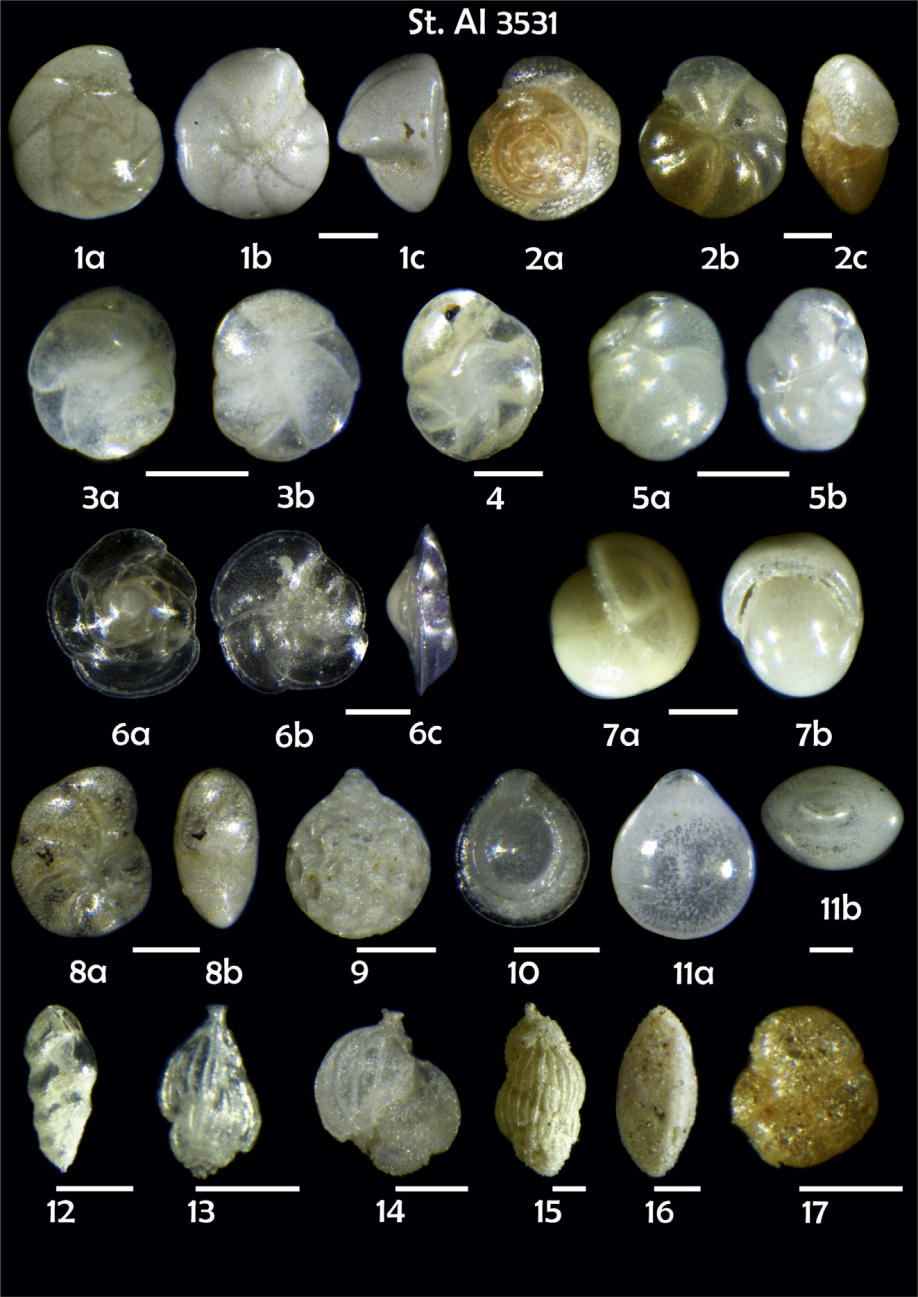
**Station 3531** **1***Hansenisca soldanii,***a** spiral view, **b** umbilical view, **c** apertural view. **2***Cibicidoides bradyi*, **a** umbilical view, **b** spiral view, **c** apertural view. **3***Cassidulina laevigata*, **a** apertural view, **b** lateral view. **4***C. teretis*. **5***Globocassidulina subglobosa*, **a**, **b** apertural view. **6***Gavelinopsis praegeri,***a** spiral view**, b** umbilical view, **c** lateral view. **7***Pullenia bulloides*, **a** side view, **b** apertural view. **8***Elphidium excavatum*, **a**, **b** side view. **9***Oolina squamosa*. **10**, **11***Fissurina* spp., **a** side view, **b** apertural view. **12***Brizalina subspinescens.***13**, **14***Trifarina angulosa*. **15***Uvigerina peregrina*. **16***Sigmoilopsis schlumbergeri*. **17***Trochammina inflata.* Scale 100 μm.

**Phototable 19:**
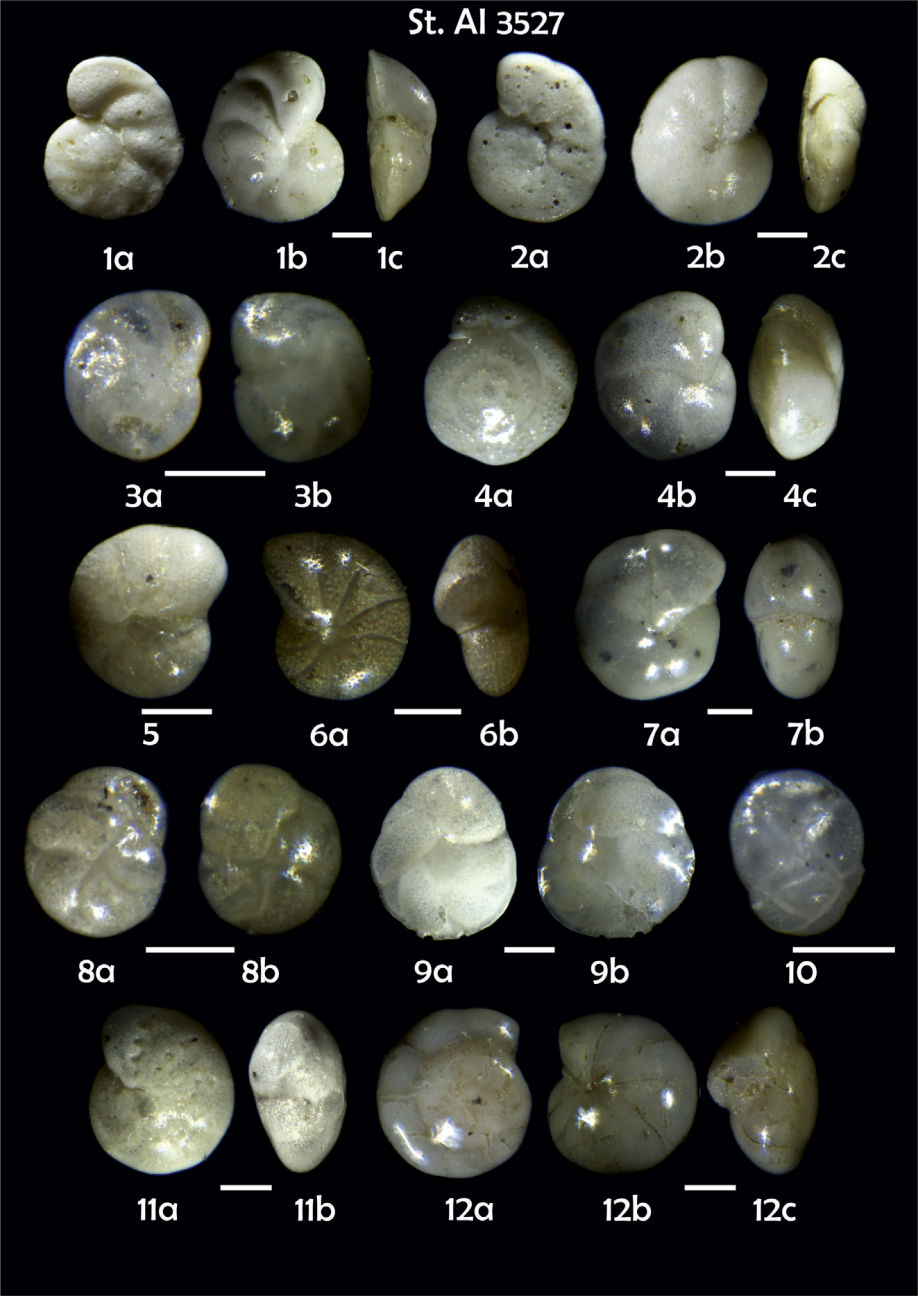

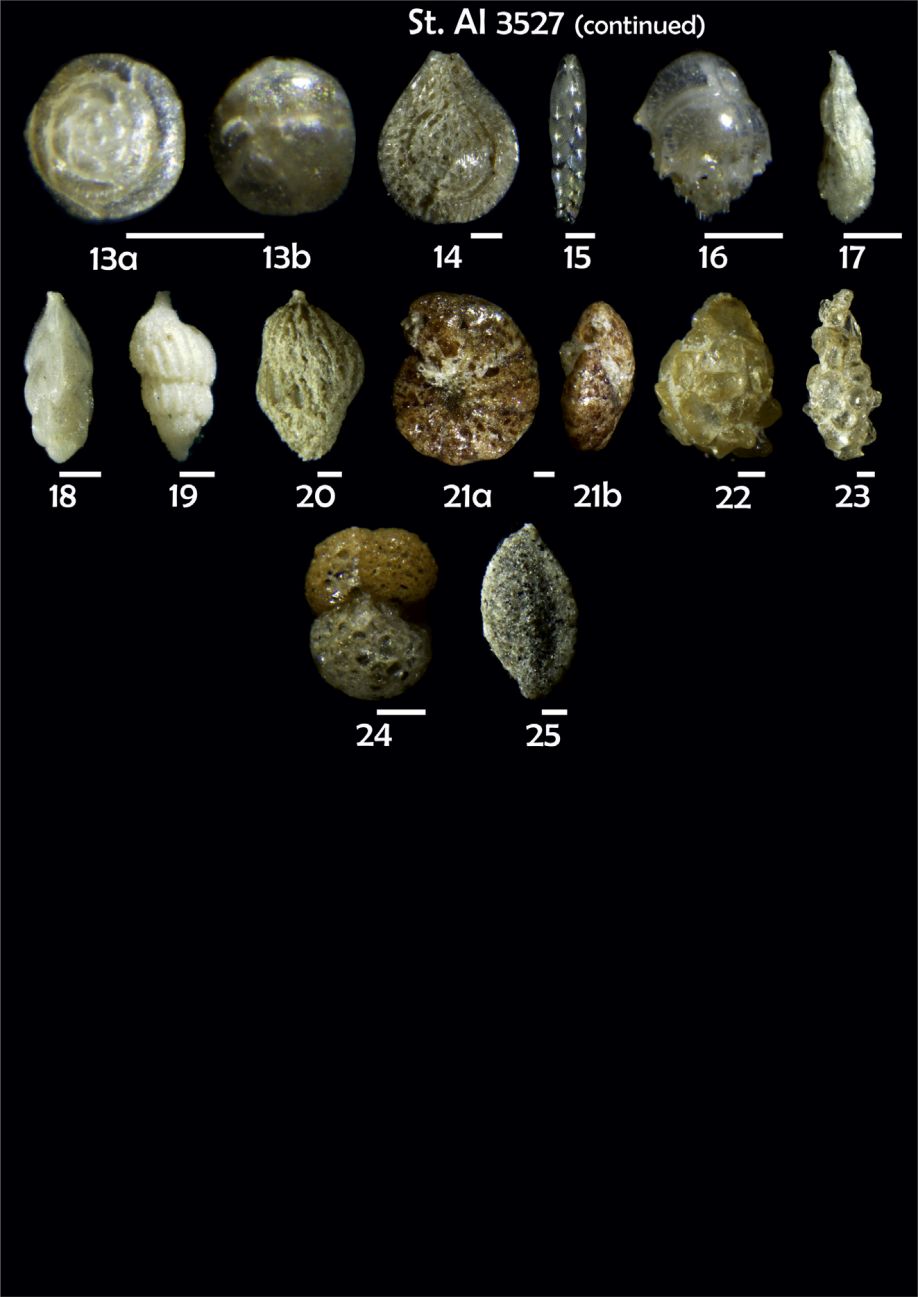
**Station 3527** **1***Cibicidoides* sp. cf. *C. lobatulus*, **a** umbilical view, **b** spiral view, **c** apertural view. **2***C. lobatulus*, **a** umbilical view, **b** spiral view, **c** apertural view. **3***C. wuellerstorfi*, **a** umbilical view, **b** spiral view. **4***C. bradyi*, **a** umbilical view, **b** spiral view, **c** apertural view. **5***Melonis* sp. **6***Melonis barleeanus,***a** side view, **b** apertural view. **7***Pullenia bulloides*, **a** side view, **b** apertural view. **8***Cassidulina reniforme*, **a** apertural view, **b** lateral view. **9***C. teretis*, **a** lateral view, **b** apertural view. **10***Globocassidulina subglobosa*. **11***Elphidium excavatum*, **a** lateral view, **b** apertural view. **12***Gyroidina orbicularis*, **a** spiral view, **b** umbilical view, **c** apertural view. **13***Alabaminella weddellensis*, **a** spiral view, **b** umbilical view. **14***Fissurina squamosoalata*. **15***Bolivina earlandi.***16***Bulimina mexicana*. **17***Trifarina angulosa*. **18***T. fluens.***19***Uvigerina* sp. **20***Uvigerina* sp. cf. *U. mediterranea.***21***Cyclammina pussila*, **a** lataral view, **b** apertural view. **22***Saccamimina sphaerica*. **23***Lagenammina difflugiformis*. **24** ?. **25***Sigmoilopsis schlumbergeri*. Scale 100 μm.

**Phototable 20:**
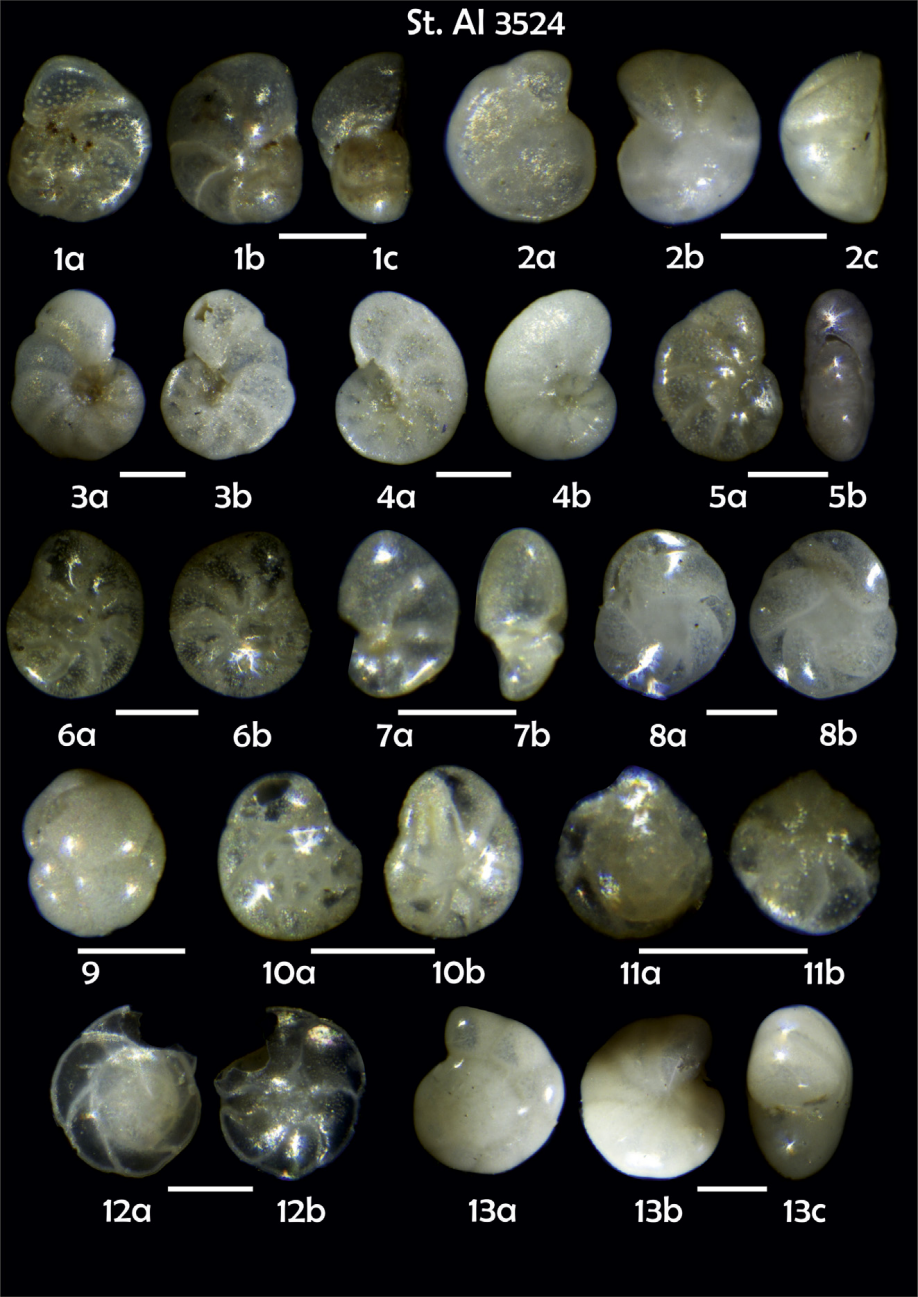

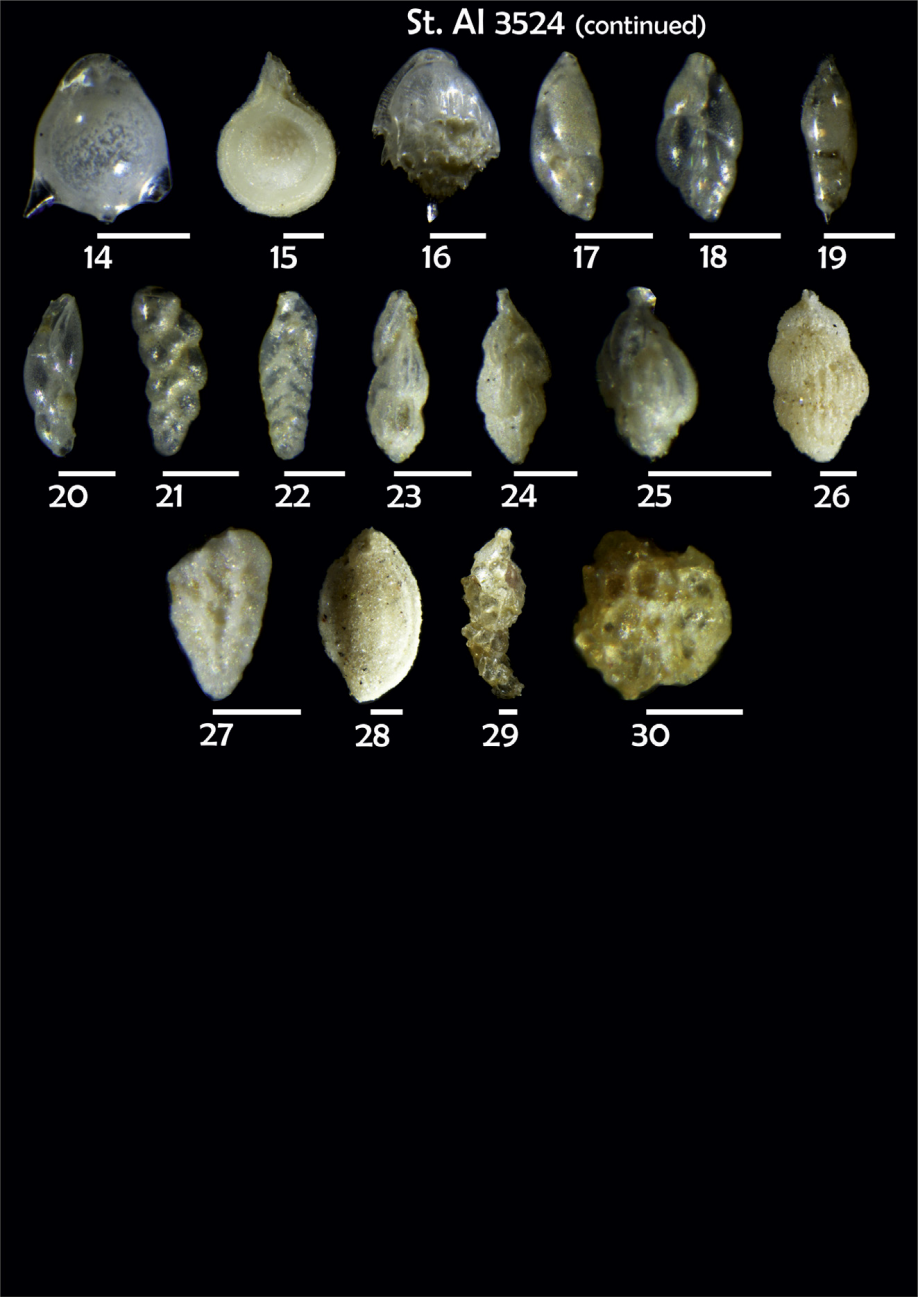
**Station 3524** **1***Cibicidoides* sp. juvenile form for *C. lobatulus*?, **a** umbilical view, **b** spiral view, **c** apertural view. **2***Cibicides refulgens*, **a** umbilical view, **b** spiral view, **c** apertural view. **3***Cibicidoides* sp., **a** umbilical view, **b** spiral view. **4***Cibicidoides* sp., **a** umbilical view, **b** spiral view. **5** ?, **a** lateral view, **b** apertural view. **6***Elphidium excavatum*, **a** umbilical view, **b** spiral view. **7***Nonionella auricula,***a** lateral view, **b** apertural view. **8***Cassidulina teretis*, **a** apertural view, **b** lateral view. **9***C. reniforme*. **10***Epistominella exigua*, **a** spiral view, **b** umbilical view. **11***Alabaminella weddellensis*, **a** spiral view, **b** umbilical view. **12***Gavelinopsis praegeri*, **a** spiral view, **b** umbilical view. **13***Oridorsalis* sp. cf. *O. umbonatus*, **a** spiral view, **b** umbilical view, **c** apertural view. **14***Fissurina staphyllearia*. **15***Fissurina* sp. **16***Bulimina mexicana*. **17**, **18***Fursenkoina fusiformis*. **19***F. pausiloculata*. **20***Fursenkoina* sp. cf. *F. complanata*. **21***Brizalina subspinescens*. **22***Bolivina* sp. **23**, **24**, **25***Trifarina angulosa*. **26***Uvegerina peregrina*. **27***Bolivina pseudoplicata*. **28***Sigmoilopsis schlumbergeri*. **29***Reophax bradyi.***30***Psammosphaera fusca*. Scale 100 μm.

**Phototable 21:**
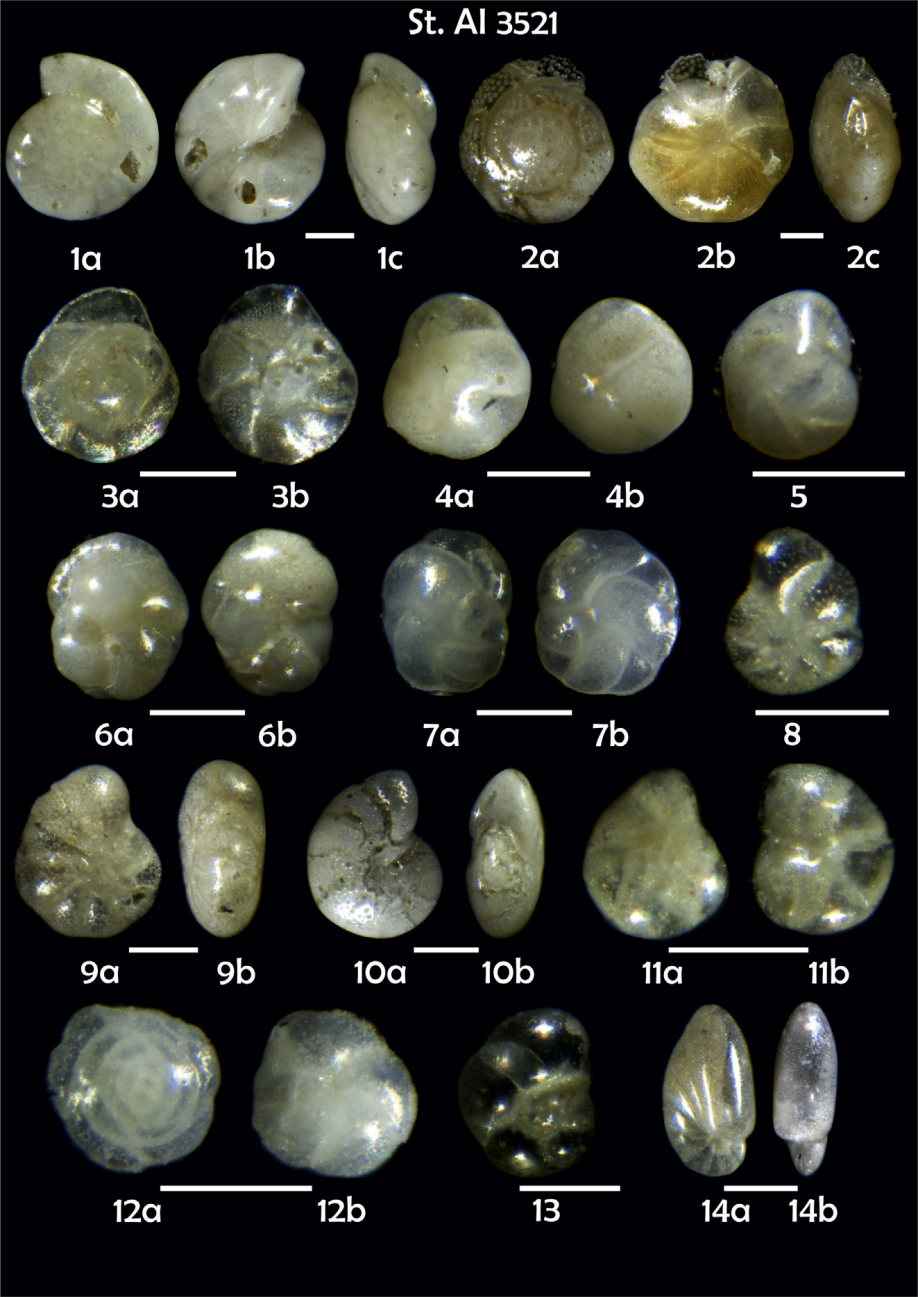

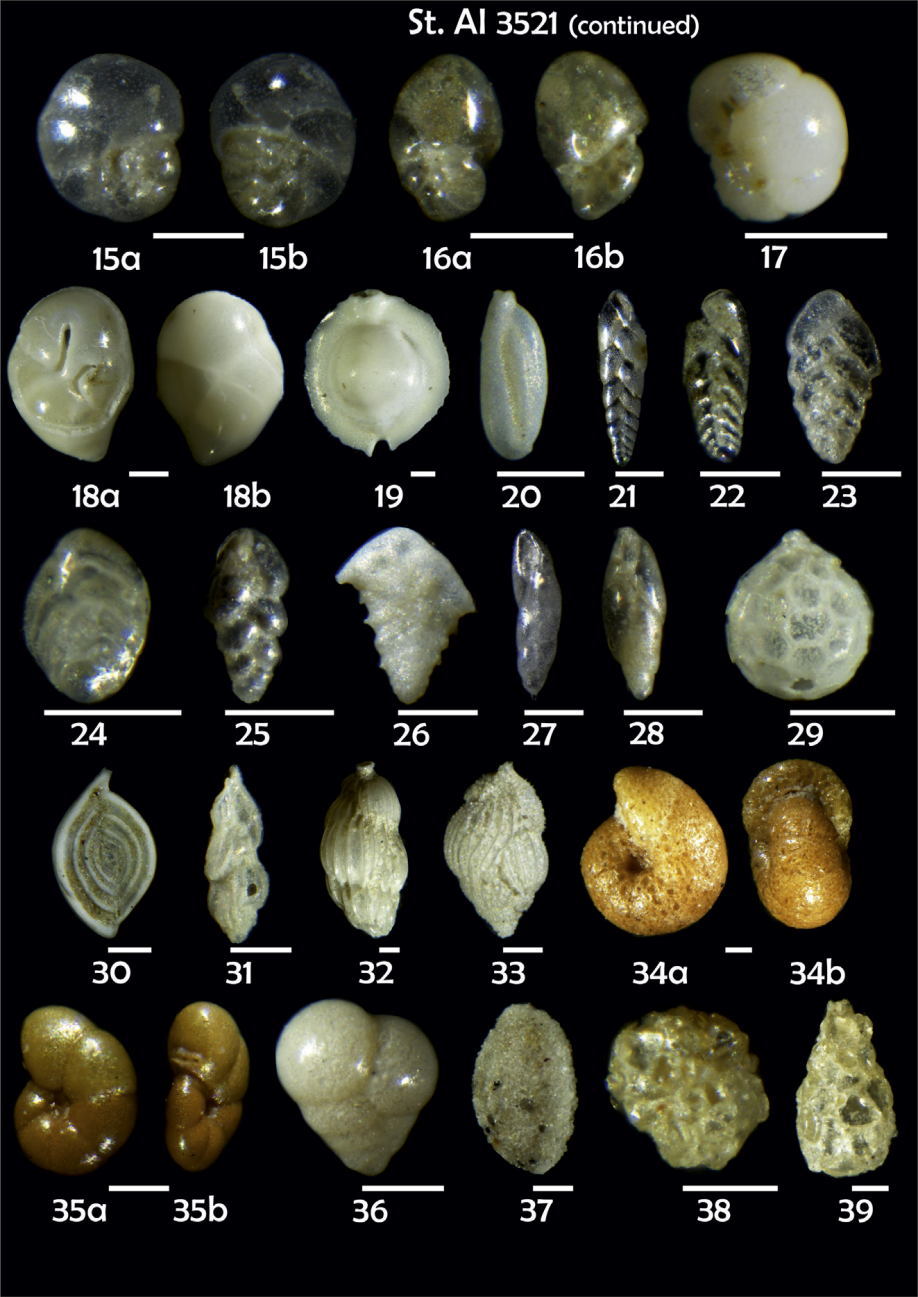
**Station 3521** **1***Gyroidina orbicularis*, **a** spiral view, **b** umbilical view, **c** apertural view. **2***Cibicidoides bradyi*, **a** umbilical view, **b** spiral view, **c** apertural view. **3***Gavelinopsis praegeri,***a** spiral view**, b** umbilical view. **4***Cassidulina laevigata*, **a** apertural view, **b** lateral view. **5***C. laevigata*. **6***C. reniforme*, **a** apertural view, **b** lateral view. **7***C. laevigata*, **a** apertutal view, **b** lateral view. **8***Elphidium subarcricum.***9***E. excavatum*?*,***a** side view, **b** apertural view. **10***Melonis*? sp. **11***Epistominella exigua,***a** spiral view, **b** umbilical view. **12***Alabaminella weddellensis*, **a** spiral view, **b** umbilical view. **13***Nonionella auricula*. **14***N. turgida*, **a** lateral view, **b** aperural view. **15***Nonionella* sp., **a** spiral view, **b** umbilical view. **16***N. auricula,***a** lateral view, **b** apertutal view. **17***Globocassidulina subglobosa*. **18***Robertinoides* sp., **a** apertural view, **b** lateral view. **19***Pyrgo murrhina.***20***Triloculina elongata*. **21***Bolivina earlandi.***22***Bolivina* sp. **23***Bolivina* sp. **24** B*olivina* sp. **25***Brizalina subspinescens*. **26***Bolivina pygmae.***27***Fursenkoina complanata*. **28***F. fusiformis*. **29***Oolina squamosa***30***Spiroloculina tenuiseptata.***31***Trifarina angulosa.***32***Uvigerina peregrina*. **33***Uvigerina* sp. **34***Cribrostomoides subglobosus*, **a** side view, **b** apertural view. **35** ?**, a** side view**, d** apertural view. **36***Eggerella bradyi*. **37***Sigmoilopsis schlumbergeri*. **38***Psammosphaera fusca*. **39***Lagenammina difflugiformis*. Scale 100 μm.

**Phototable 22:**
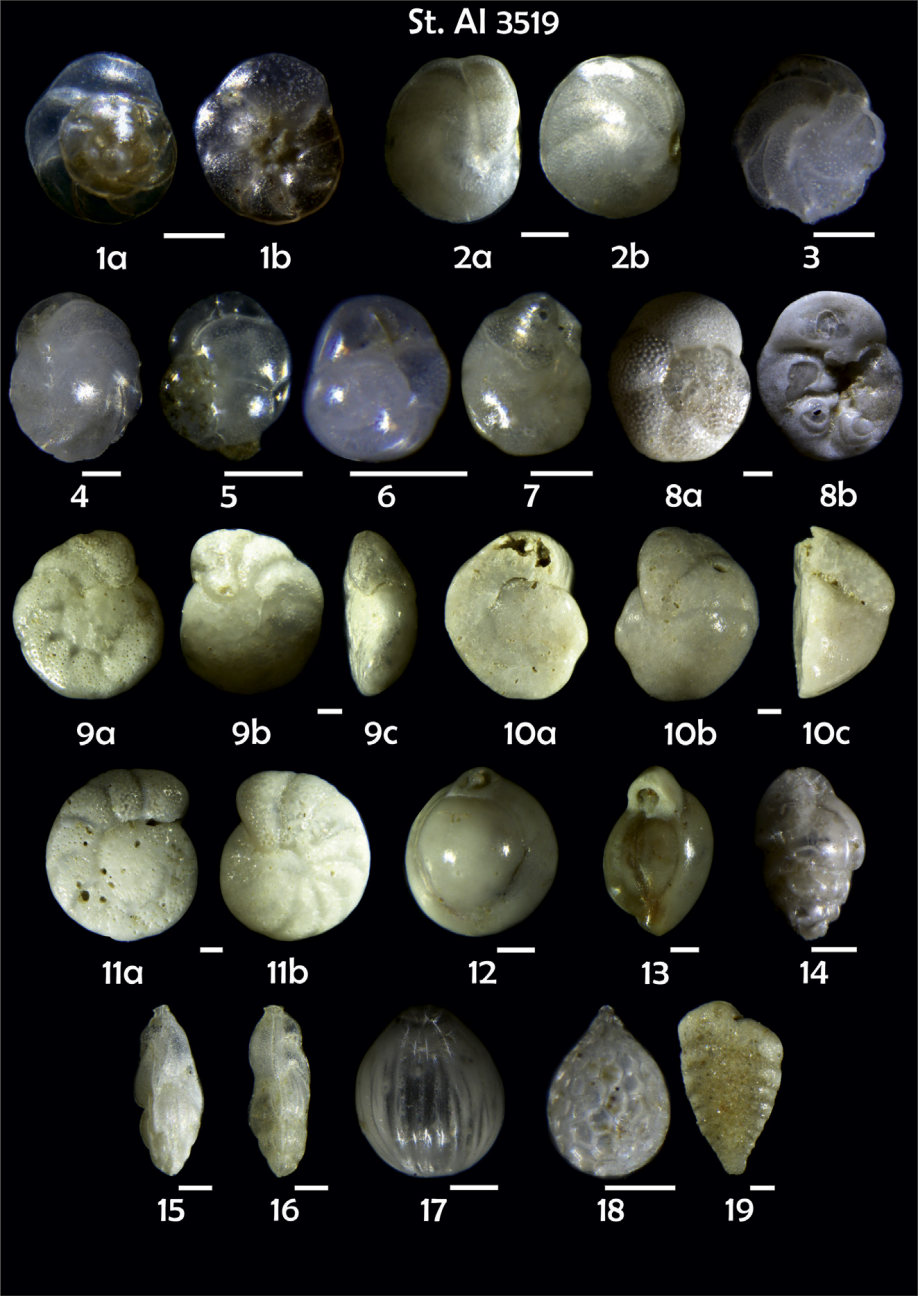
**Station 3519** **1** Rosalina vilardeboana, **a** spiral view, **b** umbilical view. **2** Cassidulina laevigata, **a** lateral view, **b** apertural view. **3** Islandiella helenae. **4** Cassidulina teretis. **5** C. reniforme. **6**, **7** Globocassidulina subglobosa. **8** Rosalina bradyi, **a** spiral view, **b** umbilical view. **9** Cibicidoides sp. cf. C. pseudoungeriana, **a** umbilical view, **b** spiral view, **c** apertural view. **10** Cibicides refulgens, **a** umbilical view, **b** spiral view, **c** apertural view. **11** Cibicidoides pachyderma, **a** umbilical view, **b** spiral view. **12** Pyrgo sp. **13** ?. **14** Bulimina marginata. **15** Trifarina angulosa. **16** T. fluens. **17** Oolina sp. **18** O. squamosa. **19** Spiroplectinella wrightii. Scale 100 μm.

**Phototable 23:**
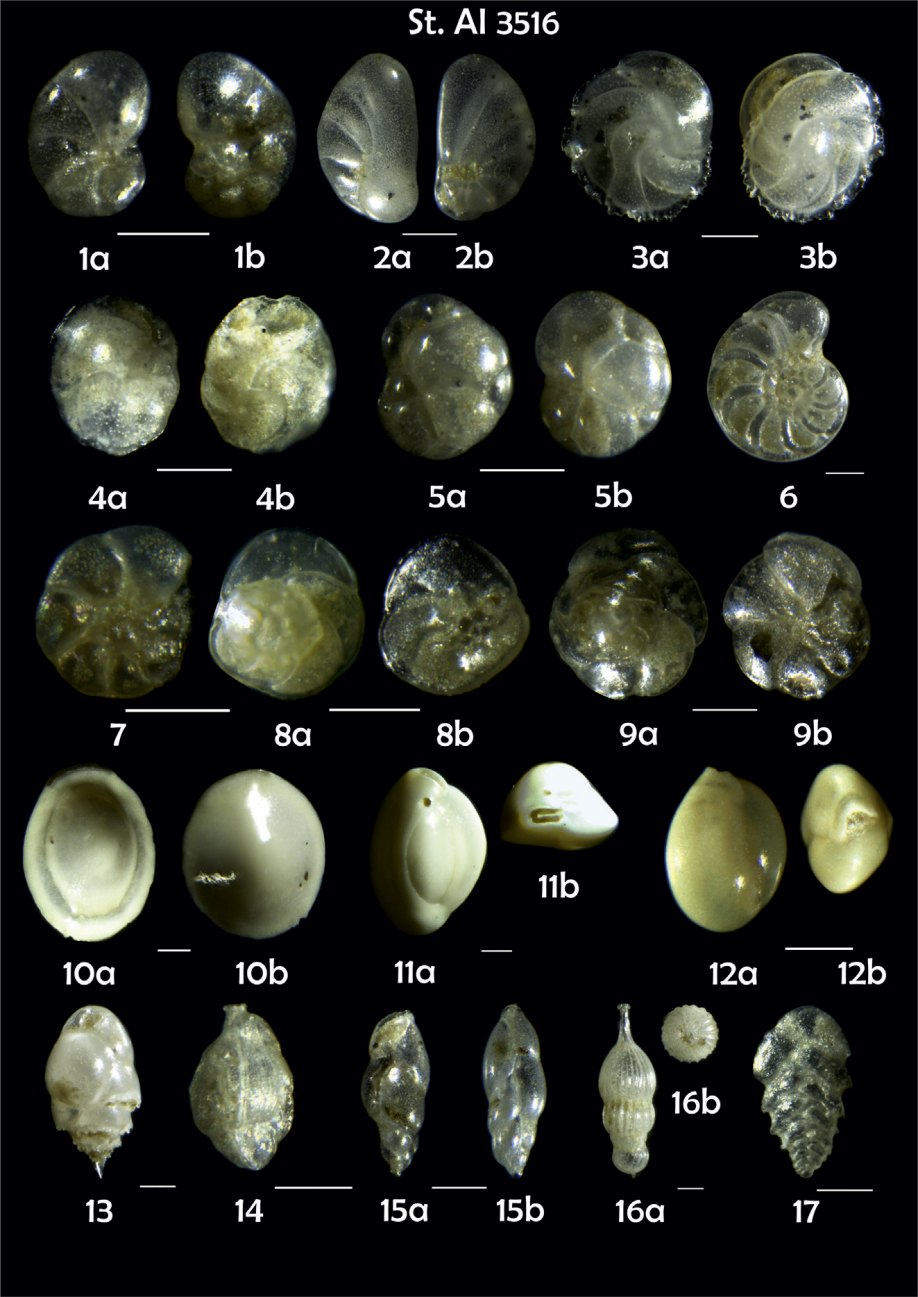
**Station 3516** **1***Nonionella auricula,***a** umbilical view, **b** spiral view. **2***N. turgida*, **a** lateral view, **b** umbilical view. **3***Cassidulina carinata*, **a** latereal view, **b** apertural view. **4***C. laevigata,***a** lateral view, **b** apertural view. **5***C. reniforme*, **a** apertural view, **b** lateral view. **6***Hyalinea balthica*. **7** ?. **8**, **9***Rosalina vilardeboana*, **a** spiral view, **b** umbilical view. **10***Pyrgo* sp. **11***Quinqueloculina seminula*, **a** lataral view, **b** apertural view. **12***Miliolinella subrotunda*, **a** lataral view, **b** apertural view. **13***Bulimina marginata*. **14***Trifarina angulosa*. **15***Fursenkoina fusiformis*. **16***Amphicoryna scalaris*, **a** lataral view, **b** apertural view. **17***Bolivina* sp. Scale 100 μm.

**Phototable 24:**
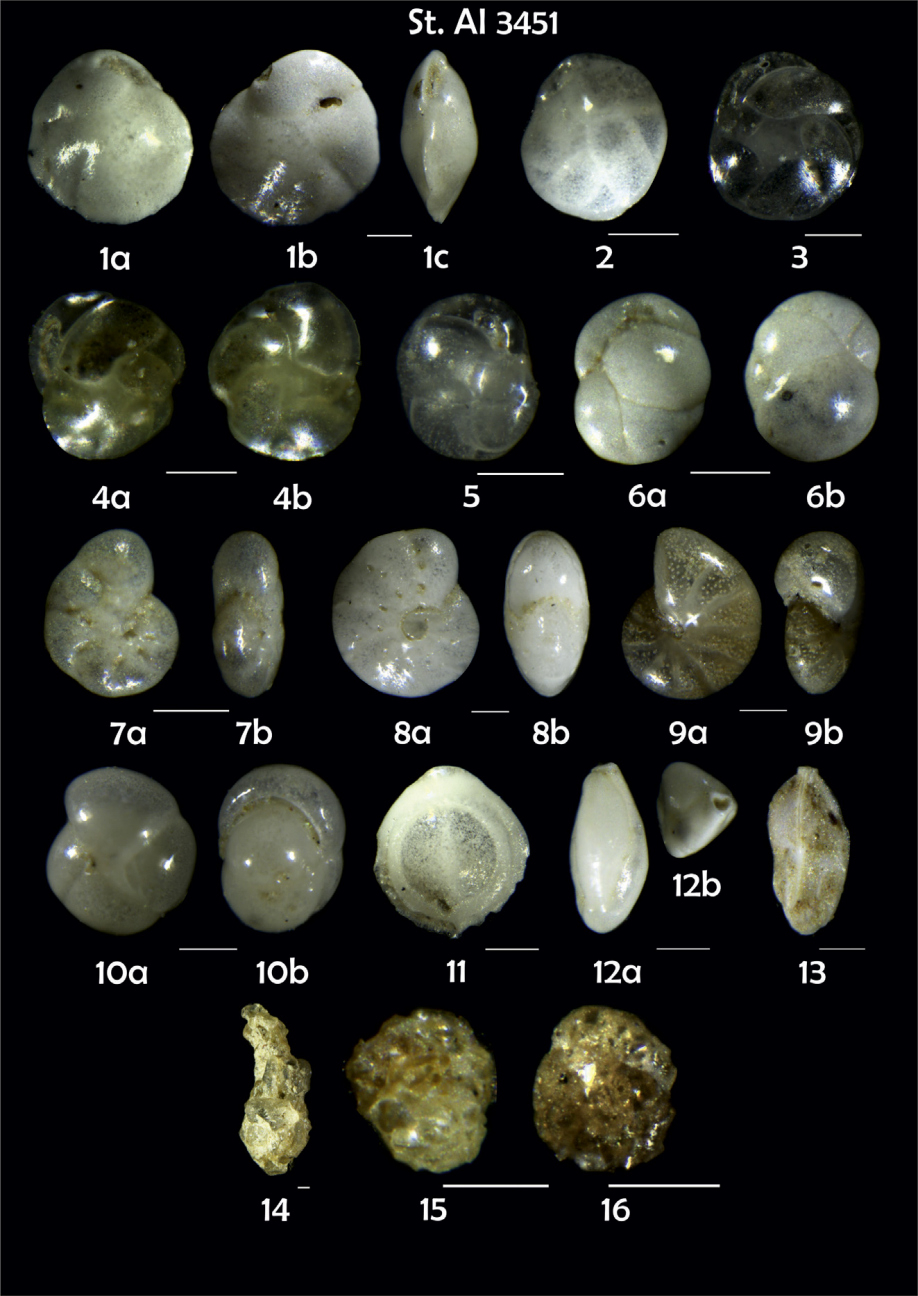
**Station 3451** **1***Cassidulina laevigata*, **a** apertural view, **b**,**c** lateral view?. 2 *C. laevigata*. **3***C. teretis*. **4***Cassidulina* sp., **a** apertural view, **b** lateral view. **5**, **6***C. reniforme*, **a** apertural view, **b** lateral view. **7***Elphidium* sp. cf. *E. excavatum*, **a** lateral view, **b** apertural view. **8***E. excavatum.***9***Melonis barleeanus,***a** side view, **b** apertural view. **10***Pullenia bulloides*, **a** side view, **b** apertural view. **11***Pyrgo* sp. ***12****Triloculina tricarinata*, **a** lateral view, **b** apertural view. **13***Trifarina angulosa*. **14***Lagenammina difflugiformis*. **15** ?. **16***Psammosphaera fusca*. Scale 100 μm.

**Phototable 25:**
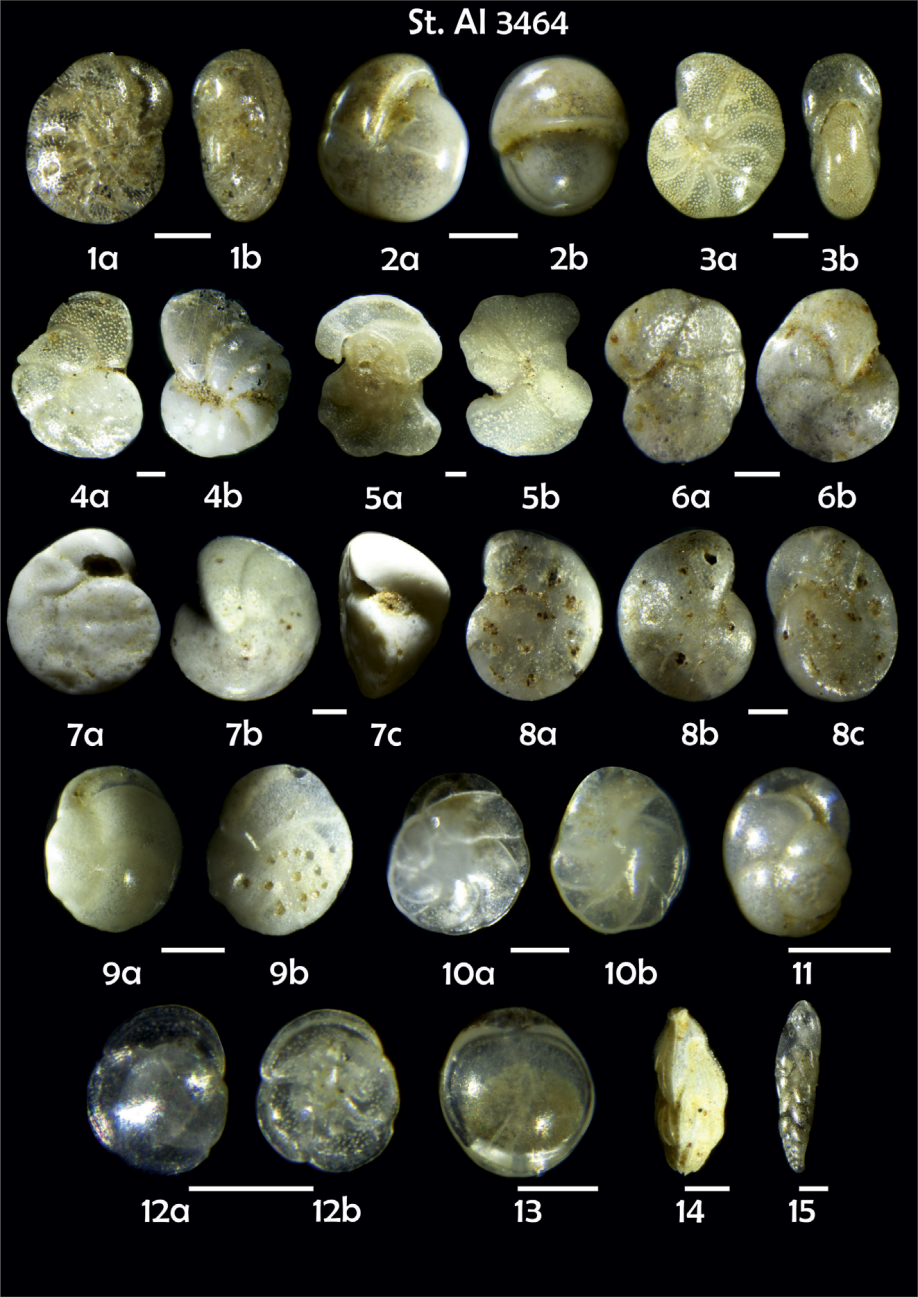
**Station 3464** **1***Elphidium excavatum*, **a** lateral view, **b** apertural view. **2***Pullenia bulloides*, **a** side view, **b** apertural view. **3***Melonis barleeanus,***a** side view, **b** apertural view. **4***Cibicidoides* sp., **a** spiral view, **b** umbilical view. **5***Cibicidoides* sp., **a** spiral view, **b** umbilical view. **6***C. lobatulus*, **a** umbilical view, **b** spiral view. **7***Cibicides refulgens*, **a** umbilical view, **b** spiral view, **c** apertural view. **8***C. wuellerstorfi*, **a** umbilical view, **b** spiral view, **c** apertural view. **9***Cassidulina laevigata*, **a** apertural view, **b** lateral view. **10***C. teretis*, **a** apertural view, **b** lateral view. **11***Globocassidulina subglobosa*. **12***Rosalina* sp., **a** spiral view, **b** umbilical view. **13***Fissurina* sp. **14***Trifarina angulosa*. **15***Bolivina earlandi*. Scale 100 μm.

**Phototable 26:**
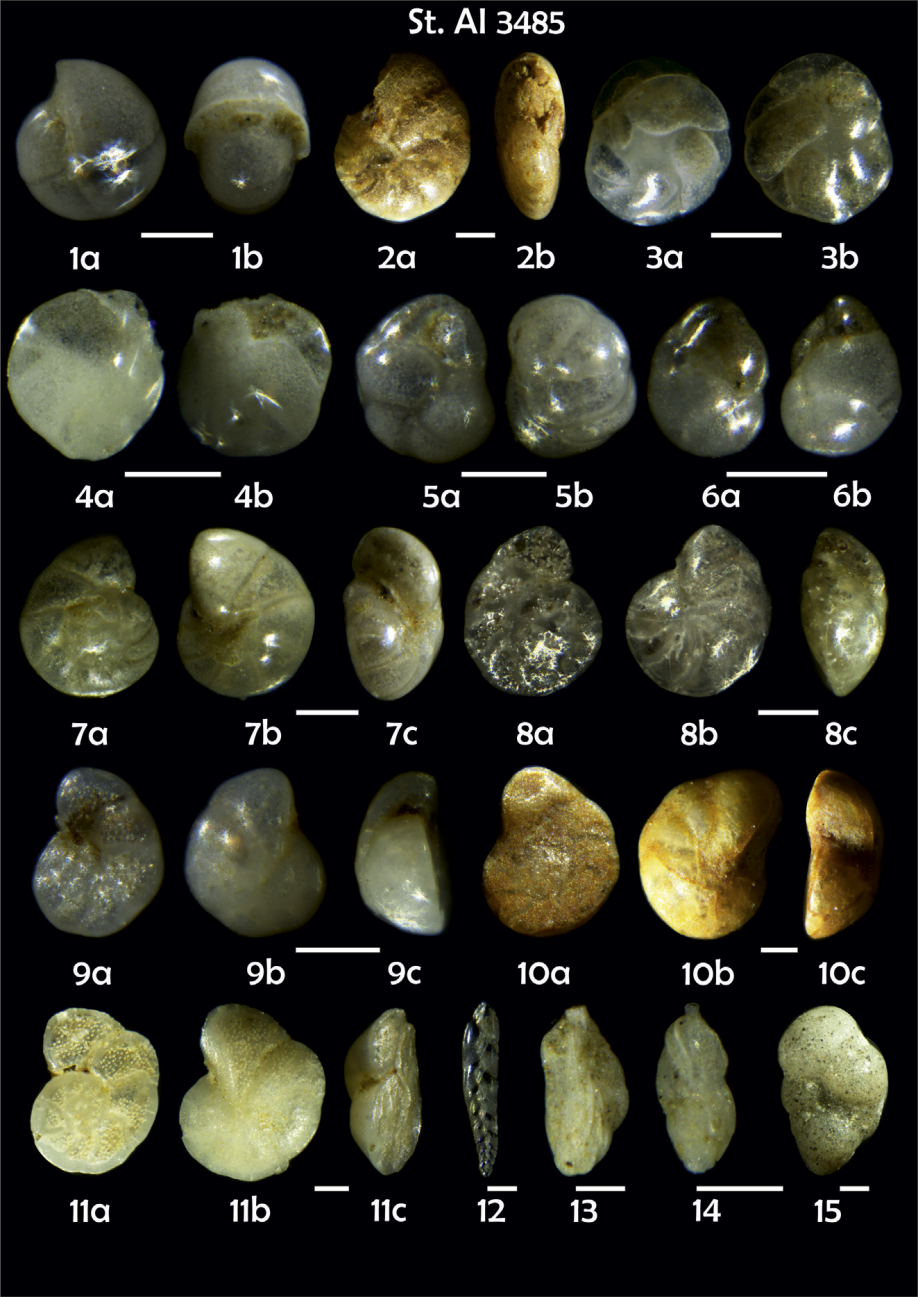
**Station 3485** **1***Pullenia bulloides*, **a** side view, **b** apertural view. **2** ?, **a** lateral view, **b** apertural view. **3***Cassidulina teretis*, **a** apertural view, **b** lateral view. **4***C. laevigata*, **a**, **b** lateral view. **5***Globocassidulina subglobosa,***a** apertural view, **b** lateral view. **6***G. subglobosa,***a** lateral view, **b** apertural view. **7***Gyroidina orbicularis*, **a** spiral view, **b** umbilical view, **c** apertural view. **8***Cibicidoides* sp., **a** umbilical view, **b** spiral view, **c** apertural view. **9, 10***Cibicides refulgens*, **a** umbilical view, **b** spiral view, **c** apertural view.**11***Cibicidoides lobatulus*, **a** umbilical view, **b** spiral view, **c** apertural view. **12***Bolivina earlandi*. **13**, **14***Trifarina angulosa*. **15***Eggerella bradyi*. Scale 100 μm.

## 2 Experimental design, materials, and methods

For the for micropaleontological analysis, we used the surface sediments (0–2 cm) from 26 grab sampler (Van-Veen type) station, obtained during the 51st cruise of the RV *Akademik Ioffe* (AI) in the summer 2016 (Fig. 1, Table 1). Then, the samples were weighed, dried and washed with distilled water through a sieve with a mesh size of 63 μm. We used a sediment fraction of 63 μm to retain such small species as *Epistominella exigua* and *Alabaminella weddellensis* [[1], [2], [3], [4]]. The identification of living foraminifera was not carried out. Further, the identification and counting was made at least 150 per one sample for benthic tests [5] (Table 2). Species definition was based on publications of Jones [6], Feyling-Hanssen [7], Atlas of Benthic Foraminifera [8], Saidova [9]. Photos were made using a Nikon microscope SMZ25 equipped with a Nikon camera DS-F*i*3 and NIS-Elements D software. Microphotograph tables were made in the computer program CorelDRAW12.

## Conflict of Interest

The authors declare that they have no known competing financial interests or personal relationships that could have appeared to influence the work reported in this paper.
